# Brain morphometry, stimulation charge, and seizure duration in electroconvulsive therapy

**DOI:** 10.1038/s41380-025-03360-y

**Published:** 2025-11-21

**Authors:** Amber M. Leaver, Chris C. Abbott, Randall T. Espinoza, Katherine L. Narr

**Affiliations:** 1 Department of Radiology, Northwestern University, Chicago, IL 60611 USA; 2 Department of Psychiatry, University of New Mexico, Albuquerque, NM USA; 3 Department of Psychiatry and Biobehavioral Sciences, University of California Los Angeles, Los Angeles, CA 90095 USA; 4 Department of Psychiatry and Behavioral Sciences, University of Washington, Seattle, WA 98102 USA; 5 Department of Neurology, University of California Los Angeles, Los Angeles, CA 90095 USA

**Keywords:** Neuroscience, Predictive markers, Depression

## Abstract

Electroconvulsive therapy (ECT) is a well-established and effective treatment for severe depression and other conditions. Yet, it is unclear why seizures are therapeutic in ECT. This study used pre-treatment brain morphology to understand why some patients need less stimulation during ECT, as well as seizure length. Pre-existing MRI data were analyzed from four cohorts with treatment refractory depression undergoing right unilateral ECT (n = 166). Pretreatment regional brain morphometry and electrical current magnitude (|E|) were analyzed, along with seizure duration and stimulation charge at seizure threshold and 6x seizure threshold. Linear models controlled for age, sex, and cohort, corrected using false discovery rate q < 0.05. Charge at seizure threshold correlated with cortical surface area perpendicular to current flow and |E| in nearby white matter, perhaps suggesting cortical involvement during seizure titration. Stimulation charge during early supra-threshold treatments correlated with cortical morphology near the right temple electrode, |E| in right amygdala and anterior hippocampus, and volume in right mid-hippocampus and thalamus. Notably, antidepressant response correlated with cortical surface area near the temple electrode and |E| in right amygdala and anterior hippocampus, the latter of which mediated the effects of the former on antidepressant response. This suggests the importance of electrical stimulation in right anteromedial temporal lobe during therapeutic seizures in ECT. Cortical surface area extending between electrodes also positively correlated with seizure duration during early treatments. Taken together, these results suggest that pre-treatment brain morphology influences ECT-induced seizure. Personalized dosing based on head morphology and other factors may improve antidepressant outcomes and reduce side effects.

## Introduction

Electroconvulsive therapy (ECT) is an effective treatment for severe or treatment-refractory depression and other conditions, in which carefully titrated electrical stimulation elicits brief, generalized tonic-clonic seizures to change the brain to improve symptoms [1]. Neuroimaging studies of ECT have shown robust and replicable brain plasticity after treatment, for example increased hippocampal volume [2–15]. Some types of neuroplasticity appear to occur most in people who respond well to ECT, typically in corticolimbic and prefrontal regions already implicated in depressive neurobiology [4, 11, 16–18]. However, it has remained unclear how seizures cause neuroplasticity leading to symptom improvement in ECT [19].

During ECT, seizure activity is monitored using 2-channel EEG to inform device settings and clinical decisions over a series of treatments [20, 21]. Seizure duration on EEG is routinely noted in the medical record, and electrical stimulus dose can be increased to improve seizure expression associated with better outcomes [20–22]. Though some studies have linked very brief seizures with greater risk for poor antidepressant outcome in ECT [23, 24], seizure duration by itself does not appear to have a strong relationship with antidepressant response [20, 25]. For example, studies testing low-amplitude or low-charge stimulation elicited seizures of typical length, but were not therapeutic [21, 26, 27]. Nevertheless, seizure duration is a widely available and used potential indicator of individual variability in seizure expression that could yield insight into how and why seizures behave differently in different people during ECT, even if its clinical utility may be unclear [20].

**Fig. 1:**
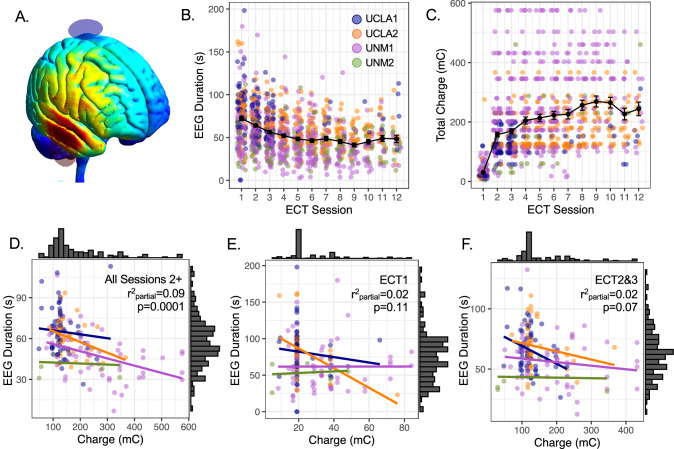
Stimulation dose and seizure duration are not strongly related during early treatment sessions. **A** Estimated electric field magnitude, |E| , is displayed for right unilateral (RUL) ECT on an MNI template head. **B**, **C** Mean seizure duration measured with EEG (sec) and mean stimulation dose in total charge (mC) are plotted over supra-threshold treatment sessions #2-#12. Error bars are standard error of the mean. **D–F** Scatterplots display relationships between seizure duration and stimulation dose during all supra-threshold sessions (D) and early treatment sessions (E-F). Color reflects study cohort, and regression lines are plotted separately for each cohort.

Variability in the electrical stimulus used to initiate seizure activity is another potential source of insight. In modern ECT, right unilateral (RUL) electrode position is typically used in a standard brief or ultra-brief pulse stimulation protocol, with one electrode near the right temple, and one electrode just to the right of the skull vertex (Fig. 1A) [28]. Pulse width and current amplitude are initially fixed, and the frequency and/or train duration of electrical pulses are varied, especially during the first session to determine individual seizure threshold, and then across subsequent sessions to maintain desired seizure expression [20]. Though each constituent part of the electrical stimulus is likely to have its own independent effects on neuronal and/or seizure activity when varied across or within patients in ECT [29], an established way to summarize stimulation dose is calculating total charge in millicolumbs (mC). Stimulation charge varies across RUL and other ECT protocols; for example, ultra-brief pulse width stimulation uses less charge on average, and may associate with fewer cognitive side effects [30]. Indeed, higher stimulation, whether delivered with higher amplitude or higher total charge, tends to associate with greater risk for cognitive side effects [27, 30–32]. Yet, within a given ECT protocol, individual variability in stimulation charge is likely to reflect how responsive each patient is to electrical seizure induction. For example, stimulation charge is associated with age and sex, with older and male patients typically requiring more stimulation [33], presumably due to differences in cortical excitability and anatomical differences (e.g., head size, skull thickness), respectively. Knowledge of how brain morphology contributes to this variability in stimulus charge could help us understand how and where seizures are initiated and/or modulated during ECT.

Relatedly, individual differences in head tissue composition and morphology determine how much electrical stimulation reaches different parts of the brain [34–37], and are also likely to impact ECT-induced seizure. In RUL ECT, estimates of electrical current magnitude, or |E|, are highest in anterior temporal cortex and inferior frontal cortex, as well as somatomotor regions under the vertex electrode [38–40]. Previous studies have reported that |E| reaching hippocampus and other brain regions correlated with the magnitude of volume increases in those same regions after ECT, though not all regions showed this pattern [40–42]. Understanding how individual variability in regional |E| relates to seizure duration and stimulation charge may also offer insight into the nature of seizure activity in ECT.

This retrospective study explored relationships between pre-treatment brain morphometry, seizure duration, and electrical stimulation in patients with depression undergoing RUL ECT. We leveraged existing datasets [2, 10, 27, 43], analyzing pre-treatment MRI data and treatment parameters collected during early ECT sessions. We hypothesized that individual variability in stimulation charge and seizure duration would correlate with morphometry and estimated |E| in regions relevant to seizure physiology, e.g., thalamus and medial temporal lobes. We targeted stimulation charge and seizure duration from the first session, where each patient’s seizure threshold is estimated using the seizure titration method [28], and during sessions two and three, where supra-threshold stimulation is delivered to elicit a therapeutic seizure. Seizure duration and stimulation charge were averaged over sessions two and three to mitigate changes in these measures across sessions for some patients [22]. Taken together, these analyses sought to use MRI to improve our understanding how seizures change the brain in ECT.

## Materials and methods

### Participants

Existing MRI data were analyzed from four separate cohorts from University of California Los Angeles (UCLA1, UCLA2) and University of New Mexico (UNM1, UNM2; Table 1). Clinical and other information can be found in source publications: [2, 10, 27, 43]. Patients (n = 166) were treatment-refractory (i.e., 2+ failed treatment attempts) and/or in need of rapid treatment, currently experiencing a major depressive episode, and gave informed written consent to participate in these studies. Additional inclusion criteria were diagnosis of major depressive disorder (UNM & UCLA) or bipolar disorder (UCLA only) using DSM-V (DSM-IV before 2013), which may include psychotic symptoms. Exclusion criteria included MRI contraindications (e.g., incompatible implants, claustrophobia), other comorbid neuropsychiatric or neurological conditions (e.g., schizophrenia, schizoaffective disorder), and concurrent serious illness. UCLA participants tapered off antidepressants, anticonvulsants, and benzodiazepines during ECT; UNM participants tapered off anticonvulsants and were permitted to remain on antidepressants and lorazepam (held evening and morning before ECT sessions). Data selection procedures are detailed in Supplemental Methods.

**Table 1:** Demographic and Clinical Information by Site.

	UCLA 1	UCLA 2	UNM 1	UNM 2
Total Sample Size	61	41	6	58
Age in years, mean(SD)	43.1 (14.1)	40.1 (15.9)	63.8 (10.9)	65.3 (8.4)
Sex, F(M)	30 (31)	26 (14)	4 (2)	44 (14)
Diagnosis, MDD(Biploar)	50 (9)	31 (10)	6(0)	58 (0)
ECT1 Seizure Duration in s, mean(SD)	81.3 (32.2)	83.2 (31.3)	53.3 (15.8)	61.8 (33.8)
ECT1 Stimulation Charge in mC, mean(SD)	22.3 (9.7)	22.6 (10.9)	23.4 (17.4)	34.1 (18.8)
Baseline HDRS, mean(SD)	23.2 (5.8)	21.5 (6.9)	24.3 (4.9)	27.7 (5.4)
Post-treatment HDRS, mean(SD)	12.4 (7.0)	15.2 (7.4)	3.2 (2.7)	13.5 (8.4)

### Ethics approval and consent to participate

Approval was obtained by the Institutional Review Boards at UNM and UCLA for their respective source studies, and informed consent was obtained from all participants for study participation and data sharing.

### ECT sessions

ECT was administered according to clinical procedures at each site and was not altered for research for UCLA1, UCLA2, and UNM1. For UNM2, participants were randomly assigned to 600, 700, 800, or 900 mA stimulation amplitude after seizure titration [27]; all other participants received 800 mA standard for RUL ECT. Apart from these differences, ECT followed similar clinical procedures, including RUL electrode positioning and ultra-brief pulse stimulation (i.e., 0.3 ms pulse width) at 6x seizure threshold at the 2^nd^ session onward, 3 sessions per week. Short-acting anesthesia (methohexital, 1 mg/kg) and a muscle relaxant (succinylcholine, 1 mg/kg) were administered during sessions, with rare exceptions (e.g., due to availability). A subset of UNM2 participants assigned to 600 mA received 1.0 s pulse width stimulation. Participants who transitioned to bitemporal electrode placement were retained for the current analyses if this transition occurred after the 3^rd^ treatment. Additional details can be found below and in Supplemental Methods.

### ECT parameters: methods and rationale

This study targeted stimulation charge and seizure duration, taken from the first ECT session (ECT1) and averaged across the 2^nd^ and 3^rd^ sessions (ECT2&3). At ECT1, stimulation parameters were recorded from the last “successful” seizure and reflected seizure threshold for each participant according to the seizure titration schedules at each site. ECT2&3 was chosen because (a) stimulation dose was supra-threshold and potentially therapeutic, (b) these sessions occurred soon after pre-treatment MRI analyzed in the current study, (c) charge and seizure duration may change over treatment course, and (d) averaging potentially reduces variability (Supplemental Methods).

We chose stimulation dose in total charge as a single measure reflecting the amount of stimulation applied during treatment. The rationale for choosing stimulation charge was to mitigate differences in amplitude across cohorts (i.e., experimentally varied in UNM2 [27] but not others), as well as variations in pulse duration and/or frequency across patients, sessions, and cohorts. Averaging charge over ECT sessions 2 and 3 also addresses any changes in these parameters across early sessions. Though each stimulation parameter comprising charge is likely to have its own unique effects on (or interactions with) brain morphometry and function [29], future prospective studies could better examine the effects of each stimulation parameter (e.g., by independently modulating each parameter in animal models as in [44, 45]).

Seizure duration was recorded at each ECT session using 2-channel EEG, used as a measure of variability in seizure expression across study participants. Though it is possible to record other seizure characteristics using ECT devices [46], and qualitative measures of seizure expression are sometimes annotated, these parameters were not recorded consistently and were considered less reliable for analysis in the current study. Seizure duration was chosen as a readily available potential indicator of individual variability in seizure expression, though we acknowledge its clinical utility may be unclear [20].

### Image acquisition & preprocessing

T1- and T2-weighted anatomical MRI were collected before treatment using a 3 T Siemens scanner at or approximately 1mm^3^ isovoxel resolution [2, 10, 41, 43]; details and sequence parameters are described in source publications [e.g., UCLA1 [2], UCLA2 [43], UNM1 [10], UNM2 [41]]. Most volunteers had both T1- and T2-weighted MRI; both were used in analyses when available. If a T2-weighted image was not available, only the T1 was used (n = 1 UCLA1, n = 1 UNM1, n = 11 UNM2).

Anatomical images were processed and morphological metrics were calculated using Freesurfer 7.2.0 [47–50]. Subcortical and white matter volumes were extracted using the standard aseg, aparc.2009s, and wmparc atlases in Freesurfer’s recon-all command [50], and add-on functions from Freesurfer calculated volumes for hippocampal subregions [51], amygdala nuclei [52], and thalamic nuclei [51]. Cortical metrics from recon-all included thickness, surface area, and curvature using the aparc.2009s atlas [47]. Statistical analyses targeted these atlas parcels across the whole brain.

Electric fields (E-fields) were estimated using SimNIBS [34, 53]. In brief, tissue segmentations derived from T1w and T2w images (gray matter, white matter, cerebrospinal fluid, skull, and scalp) were used to create tetrahedral volume conductor model or “head mesh” for each individual, and finite element models (FEMs) estimated unique patterns of current flow (E-field) between ECT electrodes in each individual head. Electrodes (5 cm diameter circles) were positioned 2 cm above the midpoint between right tragus and external canthus and 2 cm to the right of the vertex [54]. Standard recommended values for tissue conductivities were used [55]. E-field magnitude (|E|, V/m) was calculated for current amplitude used during treatment, |E|_verum_, and at the standard for ultra-brief pulse RUL ECT, 800 mA [54], |E|_standard_. Nifti |E|_verum_ and |E|_standard_ maps were registered to Freesurfer images in native space and parcellated using the same atlases as used for morphometric analyses. |E|_standard_ was selected as a standard/uniform metric reflecting the ability of the electrical stimulus to penetrate the head during RUL ECT (regardless of stimulation amplitude used), because some UNM2 patients did not receive 800 mA stimulation. Note that |E|_standard_ is similar to the E_brain_/I metric used by Lee et al. [56] and Deng, Abbott et al. [41, 57] (i.e., ratio between |E|_verum_ and electrode current, I).

Each metric type (gray matter volume, white matter volume, cortical thickness, cortical area, cortical curvature, and |E|) was harmonized across cohorts with neuroCombat [58], applied separately for each metric type in R (https://www.r-project.org). NeuroCombat uses an empirical Bayesian approach originally developed to mitigate batch effects in genomics [59], and has been successfully applied to structural and functional MRI metrics in a variety of contexts [58, 60–63].

### Statistical analyses

Statistical analyses were completed in R [64] across the whole brain using standard Freesurfer atlases (listed above) and two-tailed tests for significance. Before MRI analyses, relationships amongst ECT parameters (i.e., seizure duration, stimulation charge, and others) were analyzed. Linear regression tested relationships between seizure duration (explained variable) and stimulation charge (predictor variable) controlling for age, sex, and cohort (regressors of no interest), and vice versa (i.e., stimulation charge as explained variable and seizure duration as predictor variable). In this way, we were able to (1) assess relationships between stimulation charge and seizure duration, and (2) explore relationships between each of these variables and age and sex. We also reported pairwise correlations amongst stimulation charge and its constituent parameters (i.e., amplitude, pulse width, frequency, paired pulse train duration) separately for each cohort, because amplitude was manipulated in UNM2.

Main MRI analyses used linear regression to assess whether pre-treatment MRI statistically predicted seizure duration or stimulation charge during the first treatment (i.e., with at-threshold stimulation at ECT1) or during early therapeutic treatments (i.e., with supra-threshold stimulation at ECT2&3). In the first analysis, MRI metric was the explained variable, and predictor variables were ECT1 stimulation charge as the regressor of interest, and ECT1 seizure duration, age, sex, and cohort as additional regressors of no interest. In the second model, MRI metric was the explained variable, ECT1 seizure duration was the regressor of interest, and ECT1 stimulation charge, age, sex, and cohort were regressors of no interest. These same models were applied again using stimulation charge and seizure duration for ECT2&3 to yield four total analyses. Because seizure duration and stimulation charge may be correlated (especially in later treatments), models assessing seizure duration included stimulation charge as a nuisance regressor of no interest, and vice versa. These models were applied to each structural MRI metric calculated by Freesurfer and (separately) to |E| calculated by SimNIBS in each atlas region, corrected at p_FDR_ < 0.05 within each MRI metric type.

Statistics for main analyses of regional metrics were reported with correction for multiple comparisons using the false discovery rate (FDR) q < 0.05 (Benjamini-Hochberg method), applied separately for each model and metric type. For main analyses of |E|, we report results meeting both FDR q < 0.05 for |E|_verum_ and uncorrected p < 0.05 for |E|_standard_ (i.e., a conjunction of these two criteria), because stimulation amplitude contributes both to |E|_verum_ and modestly to stimulation charge. Statistical criterion for significance for macro-anatomical structures was uncorrected p < 0.05. Effect size is reported as partial r^2^ (partial_r2 function, [65]), an estimate of the unique variance explained by each model term.

Follow-up analyses were performed to aid interpretation for regional effect reaching these statistical criteria (Supplemental Methods). For effects of stimulation charge, analyses assessed whether effects persisted (1) when substituting charge for pulse number or amplitude and (2) when separating UNM2 and UCLA cohorts. For all effects, additional analyses determined whether each regional metric (morphometry or |E|_standard_) differed between responders ( > 50% improvement in Hamilton Depression Rating Scale, HDRS, score) and nonresponders ( < 50% improvement in HDRS or transition to bitemporal ECT). Because these analyses targeted RUL ECT, nonresponse was defined either as transition to bitemporal ECT or using the HDRS. Exploratory linear correlation and mediation analyses measured relationships amongst measures associated with antidepressant response. Additional details given in Supplemental Methods.

## Results

### Treatment parameters

Stimulation charge increased on average over sessions, tracking with decreasing seizure duration as expected (Fig. 1A-B, Figure S1, Table S1). Stimulation charge and seizure duration were correlated when averaging across all supra-threshold sessions (Fig. 1C). However, stimulation charge and seizure duration were not strongly correlated within early treatment sessions (Fig. 1E & F, Table S2), supporting their use as independent measures in MRI analyses. Seizure duration also tended to be negatively correlated with age, while charge was higher in male participants (Table S2). Follow-up analyses also showed that stimulation charge was not explained well by amplitude, but instead reflected a combination of multiple stimulus parameters in each cohort (Figure S2, Supplemental Results). Antidepressant response was also not predicted by stimulation charge or seizure duration at ECT1 or ECT2&3 (Table S3, Figure S3, Supplemental Results).

### Brain macro-volumes

Total cortical gray matter volume before treatment positively correlated with seizure duration and stimulation charge at ECT1 and ECT2&3 (p_uncorr_<0.05; Table S4). Estimated total intracranial volume before treatment also correlated with seizure duration and stimulation charge at ECT1. Subcortical gray matter volume predicted ECT2&3 stimulation charge. Effect size was largest for correlation between right cortical gray matter and seizure duration at ECT2&3, partial r^2^ = 0.062, but effect sizes were small overall for brain macro-volumes.

### Stimulation charge and regional brain morphometry

**Fig. 2:**
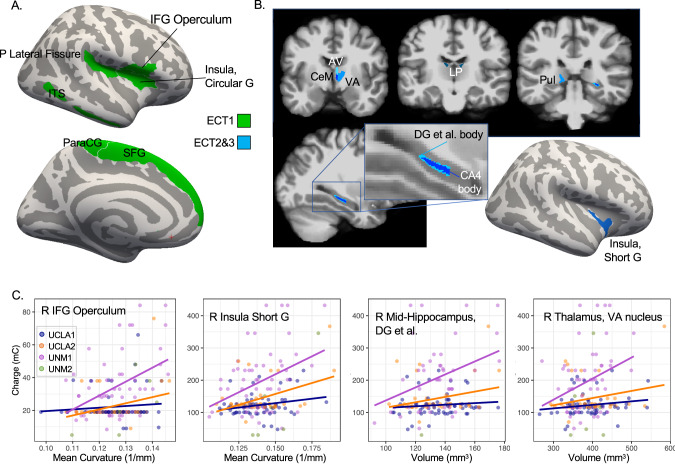
Pre-treatment regional morphometry and stimulation charge. **A** At ECT1, stimulation charge at or near seizure threshold associated with pre-treatment morphometry in cortical regions near and between electrodes (green, p_FDR_ < 0.05) displayed on template cortical surface. **B** At ECT2&3, supra-threshold stimulation charge associated with pre-treatment morphometry in thalamic nuclei, right hippocampus body, and insula (blue, p_FDR_ < 0.05). **C** Scatter plots display positive relationships between representative morphometric features and stimulation charge in mC, with separate regression lines plotted for each cohort (except UNM2, where n = 6).

ECT1 charge was statistically predicted by pre-treatment surface area on the superior surface of the right lateral fissure, including frontal operculum, circular sulcus (superior insula), and posterior lateral fissure (p_FDR_<0.05; Fig. 2, Table 2). Surface area of right inferior temporal cortex and on the left superior cortical surface (superior frontal gyrus, paracentral gyrus and sulcus) also correlated with ECT1 charge. Effect size was largest for right frontal operculum (partial r^2^ = 0.11), with full model fit (i.e., also including age, sex, and cohort) for this region explaining a larger amount of variance in ECT1 charge (adjusted r^2^ = 0.26).

**Table 2:** Regional morphometry, all pFDR < 0.05.

		Fit for brain region morphometry						Full model fit	
	Region, Measure	t	df	p	β	βse	partial r^2^	r^2^	adj r^2^
Stimulation Charge, ECT1	R Inferior Frontal G (Operculum), Curv	4.29	151	<0.0001	491.7	114.6	0.109	0.292	0.259
	R Inferior Temporal S, SA	4.21	151	<0.0001	0.029	0.007	0.105	0.290	0.257
	L Paracentral G and S, SA	3.49	151	0.0006	0.034	0.010	0.075	0.265	0.231
	R Circular S, Superior Insula, SA	3.47	151	0.0007	0.037	0.011	0.074	0.265	0.230
	R Posterior Lateral Fissure, SA	3.45	151	0.0007	0.040	0.012	0.073	0.264	0.230
	L Superior Frontal G, SA	3.27	151	0.0013	0.006	0.002	0.066	0.258	0.224
	R Inferior Frontal G Opercular Area, SA	3.15	151	0.0020	0.025	0.008	0.062	0.255	0.220
Stimulation Charge, ECT2&3	R Short Insular G, Mean Curvature	4.15	152	0.0001	1417.8	342.0	0.102	0.297	0.265
	L Thalamus, LP nucleus, Vol	3.61	152	0.0004	0.976	0.271	0.079	0.280	0.246
	R Hippocampus Body, Dentate G et al., Vol	3.25	134	0.0014	1.154	0.355	0.073	0.324	0.289
	R Thalamus, AV nucleus, Vol	3.35	152	0.0010	0.884	0.264	0.069	0.272	0.238
	R Hippocampus Body CA4 region, Vol	3.10	134	0.0024	1.289	0.416	0.067	0.319	0.284
	R Thalamus, LP nucleus, Vol	3.28	152	0.0013	0.901	0.275	0.066	0.270	0.236
	L Thalamus, Inferior Pulvinar nucleus, Vol	3.24	152	0.0015	0.576	0.178	0.065	0.268	0.235
	R Thalamus, VA nucleus, Vol	3.21	152	0.0016	0.372	0.116	0.063	0.267	0.234
	R Thalamus, Total Vol	3.15	152	0.0020	0.024	0.008	0.061	0.266	0.232
	R Thalamus CeM nucleus, Vol	3.11	152	0.0023	1.616	0.520	0.060	0.265	0.231
Seizure Duration, ECT1	R Postcentral S, SA	5.17	151	<0.0001	0.046	0.009	0.150	0.286	0.253
	R Postcentral G, SA	4.08	151	0.0001	0.057	0.014	0.099	0.243	0.208
	R Lateral Superior Temporal G, SA	3.70	151	0.0003	0.067	0.018	0.083	0.230	0.194
	R Dorsal Posterior Cingulate G, SA	3.62	151	0.0004	0.127	0.035	0.080	0.227	0.191
	R Inferior Parietal & Supramarginal G, SA	3.56	151	0.0005	0.031	0.009	0.077	0.225	0.189
	L Postcentral S, SA	3.37	151	0.0009	0.030	0.009	0.070	0.219	0.182
	L Subcallosal G, SA	3.16	151	0.0019	0.081	0.026	0.062	0.212	0.176

ECT2&3 stimulation charge was statistically predicted by right thalamus total volume and volume of anteromedial thalamic nuclei [right anteroventral (AV), right ventral anterior (VA), bilateral lateral posterior (LP), left inferior pulvinar]. ECT2&3 charge also correlated with volume of the body of the right dentate gyrus and CA4 region of the hippocampus, and curvature of short gyrus of the right insula, all p_FDR_ < 0.05 (Fig. 2, Table 2). The largest amount of variance in charge was explained by the curvature of short insular gyrus (partial r^2^ = 0.10; full model adjusted r^2^ = 0.27).

In follow-up analyses, the direction of each effect persisted for number of pulses, but not amplitude, and was more likely to reach p < 0.05 for pulse number (Table S5). In follow-up analyses for separate cohorts (UNM2, UCLA), the direction of each effect was consistent across cohorts, but was more likely to reach p < 0.05 in the UNM2 cohort, where stimulation charge range was greater (Table S6).

### Stimulation charge and regional E-field magnitude

**Fig. 3:**
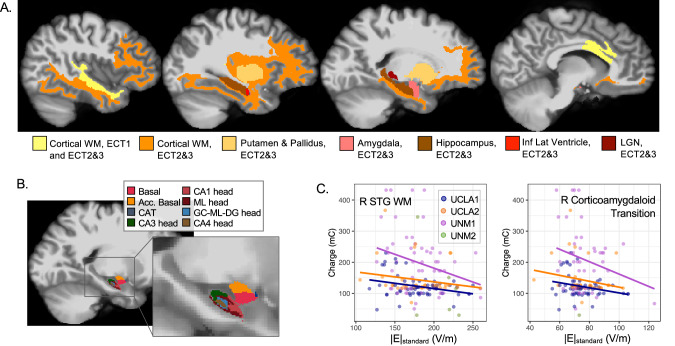
Estimated |E| and stimulation charge. **A** Regions showing relationships between stimulation charge and both |E|_verum_ p_FDR_ < 0.05 and |E|_standard_ p < 0.05 are displayed, for ECT1 and ECT2&3 in yellow and for ECT2&3 only in warm colors. Note that |E|_standard_ reflects the ability of electrical current to penetrate the head, regardless of stimulation amplitude applied, while |E|_verum_ was calculated with stimulation amplitude used during treatment. **B** Hippocampal subregions and amygdala nuclei showing relationships between supra-threshold stimulation charge (ECT2&3) and |E| are displayed; colors reflect the standard Freesurfer LUT for these atlases. **C** Scatter plots display positive relationships between |E|_standard_ in V/m and stimulation charge in mC, with separate regression lines plotted for each cohort (except UNM2, where n = 6).

ECT1 charge was statistically predicted by |E| reaching white matter in right superior and inferior temporal cortex, as well as dorsal anterior cingulate cortex and corpus callosum (|E|_verum_ p_FDR_ < 0.05, |E|_standard_ p_uncorr_ < 0.05; Fig. 3, Table 3). Effect sizes were modest, with |E|_standard_ in superior temporal white matter explaining the greatest amount of variance (partial r^2^ = 0.04; full model adjusted r^2^ = 0.21).

**Table 3:** Regional estimated |E|_standard_ and Stimulation Charge (of |E|_verum_ effects p_FDR_ < 0.05).

	Region and Measure	t	df	p	β	βse	partial r^2^	Full r^2^	Full adj r^2^
|E|_standard_, ECT1	R Superior Temporal WMV	-2.51	148	0.013	-0.09	0.04	0.04	0.24	0.21
	R Inferior Temporal WMV	-2.11	148	0.036	-0.11	0.05	0.03	0.23	0.20
	R Caudal Anterior Cingulate WMV	-2.06	148	0.041	-0.10	0.05	0.03	0.23	0.19
	R Transverse Temporal WMV	-2.03	148	0.044	-0.11	0.05	0.03	0.23	0.19
|E|_standard_, ECT2&3	R Superior Temporal WMV	-2.90	152	0.004	-0.53	0.18	0.05	0.27	0.24
	R Transverse Temporal WMV	-2.58	152	0.011	-0.67	0.26	0.04	0.26	0.23
	R Lateral Orbitofrontal WMV	-2.55	152	0.012	-0.70	0.28	0.04	0.26	0.23
	R Inferior Temporal WMV	-2.51	152	0.013	-0.63	0.25	0.04	0.26	0.23
	R Caudal ACC WMV	-2.49	152	0.014	-0.61	0.25	0.04	0.26	0.23
	R Insula, WMV	-2.46	152	0.015	-0.71	0.29	0.04	0.26	0.23
	R Inferior Frontal (Pars Triangularis) WMV	-2.40	152	0.017	-0.55	0.23	0.04	0.26	0.22
	R Entorhinal WMV	-2.34	152	0.021	-0.73	0.31	0.03	0.26	0.22
	R Parahippocampal WMV	-2.31	152	0.022	-1.01	0.44	0.03	0.26	0.22
	R Inferior Frontal (Pars Orbitalis) WMV	-2.31	152	0.022	-0.56	0.24	0.03	0.26	0.22
	R Rostral Middle Frontal WMV	-2.25	152	0.026	-0.63	0.28	0.03	0.26	0.22
	R Middle Temporal WMV	-2.25	152	0.026	-0.49	0.22	0.03	0.25	0.22
	R Fusiform WMV	-2.18	152	0.031	-0.86	0.40	0.03	0.25	0.22
	R Inferior Frontal (Pars Opercularis) WMV	-1.98	152	0.050	-0.43	0.22	0.03	0.22	0.18
	R Amygdala, CAT Volume	-2.79	135	0.006	-1.44	0.52	0.05	0.33	0.29
	R Amygdala, Total Volume	-2.94	152	0.004	-1.37	0.47	0.05	0.27	0.24
	R Amygdala, AB Volume	-2.54	135	0.012	-1.37	0.54	0.05	0.32	0.29
	R Amygdala, Basal Volume	-2.47	135	0.015	-1.30	0.53	0.04	0.32	0.28
	R Amygdala, Paralaminar Volume	-2.33	135	0.021	-1.21	0.52	0.04	0.32	0.28
	R Hippocampus Head, HATA Volume	-2.78	135	0.006	-1.33	0.48	0.05	0.33	0.29
	R Hippocampus, Parasubiculum Volume	-2.49	135	0.014	-1.44	0.58	0.04	0.32	0.28
	R Hippocampus, Total Volume	-2.55	152	0.012	-1.20	0.47	0.04	0.26	0.23
	R Hippocampus Head, Dentate et al. Volume	-2.39	135	0.018	-1.22	0.51	0.04	0.32	0.28
	R Hippocampus Head, CA3 Volume	-2.38	135	0.019	-1.20	0.51	0.04	0.32	0.28
	R Hippocampus Head, CA4 Volume	-2.20	135	0.029	-1.09	0.49	0.03	0.31	0.28
	R Hippocampus Head, CA1 Volume	-2.10	135	0.037	-1.04	0.49	0.03	0.31	0.28
	R Hippocampus Head, Molecular Layer Volume	-2.03	135	0.044	-0.96	0.47	0.03	0.31	0.27
	R Thalamus, Lateral Geniculate Nucleus Volume	-2.15	152	0.033	-0.80	0.37	0.03	0.25	0.22
	R Putamen Volume	-2.58	152	0.011	-1.05	0.41	0.04	0.26	0.23
	R Globus Pallidus Volume	-2.52	152	0.013	-0.89	0.35	0.04	0.26	0.23
	Pons Volume	-2.05	152	0.042	-1.45	0.71	0.03	0.25	0.22
	R Inf Lateral Ventricle Volume	-2.54	152	0.012	-0.84	0.33	0.04	0.26	0.23

Stimulation charge during ECT2&3 was statistically predicted by estimated |E| reaching several right amygdala nuclei and anterior hippocampal subregions (Fig. 3). Stimulation charge during ECT2&3 was also associated with |E| in right putamen, globus pallidus, and white matter in right temporal, prefrontal, and insular cortex measured before treatment (|E|_verum_ p_FDR_ < 0.05, |E|_standard_ p_uncorr_ < 0.05; Table 3). Effect sizes were modest, with |E|_standard_ reaching the right corticoamygdaloid transition explaining the greatest amount of variance in stimulation charge (partial r^2^ = 0.05; full model adjusted r^2^ = 0.29).

In follow-up analyses, the direction of effects from the main analysis of |E|_standard_ persisted for number of pulses, but not amplitude, and were more likely to reach p < 0.05 for number of pulses (Table S7). The direction of each effect also remained consistent across cohorts, but was more likely to reach p < 0.05 in the UCLA cohort, where a single amplitude (800 mA) was delivered across more participants (Table S8).

### Seizure duration, regional brain morphometry, and E-field magnitude

**Fig. 4:**
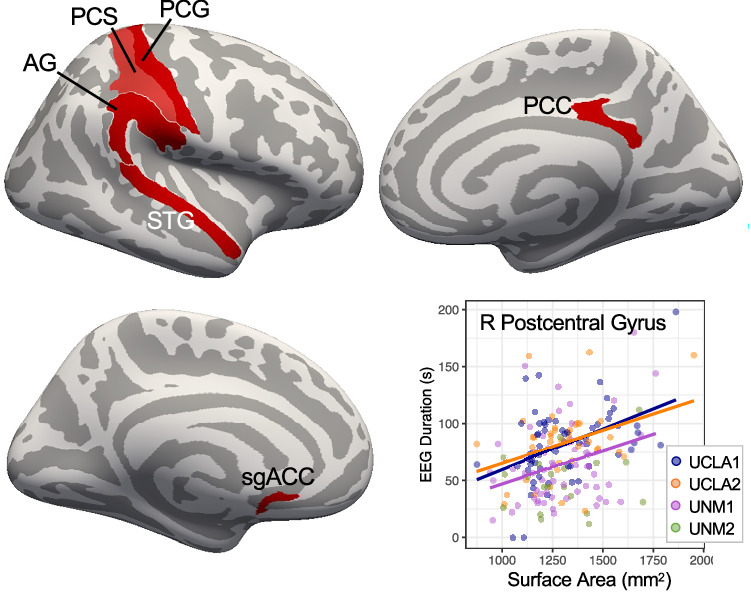
Pre-treatment cortical surface area between RUL electrodes correlates with seizure duration at first ECT session. Regions showing significant correlation (p_FDR_<0.05) between morphometry and seizure duration (ECT1) are displayed on template cortical surfaces (red). Scatter plot at bottom right displays positive relationship between a representative morphometric feature and seizure duration, with separate regression lines plotted for each cohort (except UNM2, where n = 6).

ECT1 seizure duration was statistically predicted by surface area of lateral cortical regions between RUL electrodes before treatment, including right postcentral gyrus and sulcus, and right lateral superior temporal, inferior parietal, and supramarginal gyri, p_FDR_ < 0.05 (Fig. 4, Table 2). Surface area of left subcallosal cingulate gyrus, right dorsal posterior cingulate gyrus, and left postcentral sulcus also positively correlated with ECT1 seizure duration. Effect size was largest for right postcentral sulcus area (partial r^2^ = 0.15, full model adjusted r^2^ = 0.25). When analyzing ECT2&3 seizure duration, no relationships with regional brain morphometry were found. No relationships were found between seizure duration and |E|.

### Relationships with antidepressant response to RUL ECT

**Fig. 5:**
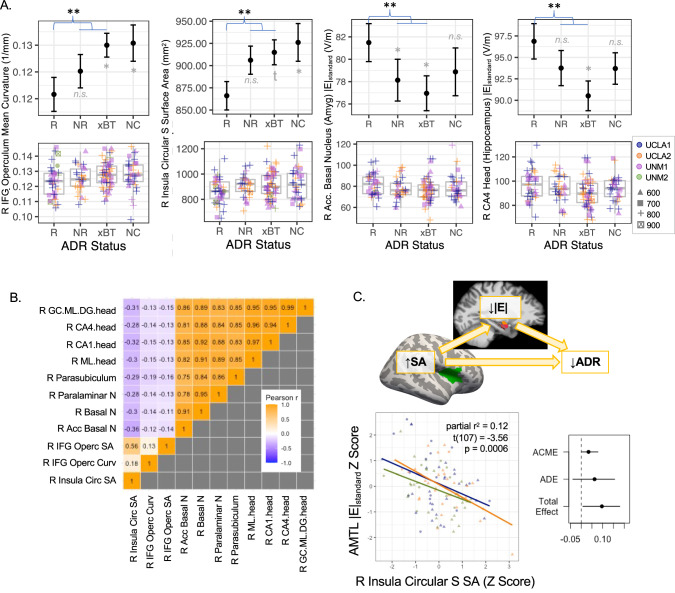
Pre-treatment morphometry and |E|_standard_ differed between people who responded to RUL ECT and nonresponders (including transition to BT ECT). **A** Plots are displayed for representative regions showing effects of response p < 0.05. In top row, mean and standard error are plotted for responders (R, >50% HDRS improvement), nonresponders (NR, <50% HDRS improvement), transition to bitemporal ECT (xBT), and non-completers (NC). In second row, boxplots are displayed, along with datapoints for each participant, with color indicating cohort and shape amplitude. **B** Cross-correlation and heat map are displayed for all regional metrics showing effects of response p < 0.05. **C** Scatter plot displays opposing relationship between |E|_standard_ in basal nucleus of the amygdala and right anterior insula surface area. Schematic of mediation analysis is displayed at top, and mean effects are plotted for average causal mediated effect (ACME), average direct effect (ADE), and total effect; error bars are 95% confidence intervals.

Follow-up analyses measured whether regions identified in the main analyses differed in people who responded to RUL ECT compared with nonresponders (Fig. 5, Table 4, Table S9). Here, responders had less surface area on average in right anterior insula (circular sulcus) and frontal operculum, and greater curvature in right frontal operculum. |E|_standard_ was also higher for responders in anterior hippocampus and select amygdala nuclei.

**Table 4:** Main analysis effects showing differences in responders to RUL ECT, p < 0.05.

	Region and Measure	t	df	p	β	βse	partial r^2^
Morph x Charge ECT1	R Inferior Frontal G Opercular Area, SA	2.58	117	0.011	64.66	25.08	0.05
	R Circular S, Superior Insula, SA	2.01	117	0.047	38.211	18.991	0.033
	R Inferior Frontal G Opercular Area, Mean Curv.	2.80	117	0.006	0.005	0.002	0.063
|E|_standard_ x Charge ECT2&3	R Amygdala, Paralaminar Volume	-3.02	107	0.003	-6.834	2.261	0.079
	R Hippocampus, Parasubiculum Volume	-2.75	107	0.007	-5.558	2.019	0.066
	R Hippocampus Head, Molecular Layer Volume	-2.73	107	0.007	-6.830	2.499	0.065
	R Amygdala, Basal Volume	-2.69	107	0.008	-5.927	2.201	0.063
	R Hippocampus Head, CA1 Volume	-2.64	107	0.009	-6.420	2.427	0.061
	R Hippocampus Head, CA4 Volume	-2.27	107	0.025	-5.495	2.424	0.046
	R Hippocampus Head, Dentate et al. Volume	-2.16	107	0.033	-5.044	2.340	0.042
	R Amygdala, AB Volume	-1.97	107	0.051	-4.095	2.079	0.035

Examining these regions that differed between responders and nonresponders to RUL ECT, positive pairwise correlations were noted in |E|_standard_ amongst right anteromedial temporal lobe (AMTL) regions (e.g., amygdala nuclei, anterior hippocampal subregions) and in surface area amongst cortical regions. An opposing relationship was also noted between right anterior insula surface area and right AMTL |E|_standard_ (Fig. 5), such that participants with greater right AMTL |E|_standard_ tended to have less right anterior insula surface area. In a follow-up mediation analysis, mean AMTL |E|_standard_ partially mediated (explained) the relationship between anterior insula surface area and antidepressant response (mediated effect or ACME = 0.03, 95% CI = [0.001, 0.08], p = 0.04).

## Discussion

This study analyzed relationships between pre-treatment brain morphology, seizure duration on EEG, and the amount of stimulation required (or chosen) during RUL ECT in participants experiencing a severe, treatment-refractory major depressive episode. When stimulation was at seizure threshold (ECT1), stimulation charge correlated with surface area of cortical tissues separating RUL electrodes (i.e., perpendicular to current flow), as well as estimated magnitude of electrical current (|E|) in nearby cortical white matter. When supra-threshold doses were delivered (ECT2&3), stimulation charge correlated with tissue volume in subcortical structures, including thalamic nuclei implicated in seizure generalization and termination [66–68] and mid-hippocampus implicated in antidepressant response to ECT [69, 70]. ECT2&3 charge also correlated with |E| reaching seizure-genic regions in right anteromedial temporal lobe (AMTL) like amygdala [71, 72], which was partially constrained by cortical surface area near the right temporal electrode and related to antidepressant response. ECT2&3 seizure duration correlated with cortical surface area extending between RUL electrodes (i.e., parallel to electrical current flow), suggesting that stimulating more neurons may lead to modestly longer seizure [73]. Taken together, these results demonstrate that pre-treatment brain morphology impacts ECT-induced seizure. Improved mechanistic understanding of ECT-induced seizures, and particularly seizure variability across patients and sessions, could help develop approaches to precision dosing and other improvements to ECT.

### Subcortical vs. cortical involvement in ECT-induced seizure

Stimulation amplitude, and therefore |E|, typically does not change between seizure titration and subsequent sessions. However, the number, width, and/or frequency of electrical pulses (and therefore stimulation charge) delivered across the |E| distribution does change. In our study, ECT1 charge correlated exclusively with cortical metrics, while ECT2&3 charge correlated with subcortical metrics. Though qualitative, this difference is notable given that each pulse delivered also likely exceeds the threshold for neuronal firing in a majority of brain tissue [57, 74]. One possible interpretation is that seizure initiation occurs primarily in right cortical regions when stimulation is at seizure threshold and pulse trains are shorter (though subcortical structures like thalamus are almost certainly recruited during these generalized seizures [75]), while subcortical regions like thalamus, hippocampus, and amygdala become more relevant to ECT-induced seizure during supra-threshold stimulation, i.e., when pulse trains are longer. Correlations noted between ECT2&3 pulse number and |E|_standard_ or morphometry in subcortical regions also support this idea. Given the seizure-genic nature of amygdala and nearby anteromedial temporal lobe regions [71, 76], we speculate that |E| reaching anteromedial temporal lobe (AMTL) regions may only be sufficient to initiate seizure activity in AMTL during supra-threshold sessions, where longer pulse trains are applied. However, empirical studies directly measuring the distribution of seizure activity as it unfolds during at- and supra-threshold ECT with adequate spatial and temporal resolution are needed to confirm this interpretation.

If the site(s) of seizure initiation differ for at- versus supra-threshold dosing in RUL ECT, this could have implications for the seizure titration method as a means of determining threshold. For example, if antidepressant outcome in RUL ECT relies on the brain’s response to seizure activity initiated in right AMTL, more precisely matching seizure threshold in right AMTL in each patient could improve outcomes (i.e., versus matching seizure threshold in right cortical regions, presumably as in current seizure titration methods). Conversely, if the location(s) of seizure initiation is not relevant to antidepressant response to RUL ECT, then perhaps a more general measure of cortical excitability could offer an equally accurate but less invasive/burdensome means to estimate seizure threshold (e.g., TMS-induced MEPs, or a functional neuroimaging-based biomarker). Future studies could consider these issues to improve seizure threshold estimation, to more precisely determine dose without seizure and improve outcomes.

ECT2&3 charge also correlated with pre-treatment anteromedial thalamic and mid-hippocampal volume but not |E|, so perhaps these effects are more relevant to seizure generalization than initiation. The thalamus is well-known to play a role in seizure generalization and modulation [66, 75] [77–83], and thalamic deep brain stimulation can be effective in treating refractory epilepsy [67, 68]. ECT-MRI studies also report correlation between increased thalamic CBF after the first supra-threshold session and later antidepressant outcome (UCLA1 [69]), as well as thalamic plasticity after ECT [84, 85]. Changes in mid-hippocampal function also associate with antidepressant response in ECT (UCLA1 [69, 70]), and connections between hippocampus and anteromedial thalamus are thought to be involved in modulating hippocampal seizure [86, 87]. Future studies examining connectivity amongst these regions in relation to ECT2&3 charge or seizure expression (e.g., ictal theta [88]) could be informative in predicting susceptibility to generalized seizure in ECT.

### Cortical morphology, current flow, and antidepressant response to ECT

In this study, ECT1 charge positively correlated with the size of cortical surfaces extending perpendicular to electrical current flow. This could suggest that patients with larger cortical surfaces obstructing the flow of electrical current between electrodes required more stimulation to elicit seizures during titration, perhaps due to greater shunting of electrical current in less-resistive CSF across cortical surfaces perpendicular to current flow [89]. This interpretation is also supported by our finding that patients with less |E| penetrating nearby right cortical white matter also tended to require greater ECT1 charge (perhaps) to compensate. Taken together, these results demonstrate how the size (and/or shape) of cortex near electrodes may constrain stimulus delivery to key regions during ECT.

Anterior insula surface area near the right temporal electrode also appeared to influence how much stimulation reached right amygdala and anterior hippocampus (i.e., AMTL |E|_standard_), both of which also associated with antidepressant response to RUL ECT. Here, nonresponders (including transition to BT ECT) tended to have larger right insula surface area and less |E|_standard_ in right amygdala and anterior hippocampus. Though previous studies reported finding no relationship between antidepressant response and hippocampal or amygdalar |E| [40, 41], these studies did not analyze hippocampal subregions or amygdala nuclei and/or include transition to BT ECT as nonresponse to RUL ECT. Our results suggest that adjusting dose to increase |E| in right AMTL (e.g., greater amplitude, more pulses, or adjusting electrode position) could improve outcomes for some patients in RUL ECT. However, it is also important to note that effect sizes were relatively modest, and other factors are likely to play a role in ECT-induced seizure, antidepressant response, and stimulus delivery (e.g., skull thickness).

### Morphological predictors of seizure duration

Patients with longer seizure duration at ECT2&3 tended to have more cortical surface area extending between RUL electrodes, including right postcentral sulcus and gyrus, superior temporal gyrus, and adjacent parietal regions. These same brain regions extend parallel with the direction of current flow, and also tended to receive greater |E| than elsewhere in the brain (Fig. 1A; [37, 38, 73]). Cortical surface area is linked with a number of ontological/developmental processes [90, 91], and could reflect the number or size of cortical columns within a given region [92, 93]. Thus, the present results could suggest that the number neurons maximally stimulated during pulsed electrical current may contribute to seizure length during typical RUL treatment sessions [73]. Also of note, subgenual and posterior cingulate were associated with seizure duration but not antidepressant response, despite their roles in depressive neurobiology [94–96] (Supplemental Discussion).

These results also suggest that brain morphology could inform statistical models predicting seizure length; the combination of age, sex, and right precentral sulcus area explained 25% of the variance in seizure duration, a large effect size (Table 2). The utility of such models in predicting antidepressant response remains unclear [20, 25, 97] – some studies have linked very brief seizures with greater risk for poor antidepressant outcome [23, 24], while others report cases where very brief seizures were therapeutic [20]. However, predictive models that identify risk for long seizures associated with side effects [98] could be useful. Yet it is important to note that seizures were of typical length in this study, and different aspects of brain morphology and/or function may better explain seizure length (e.g., measures of neuronal excitability, connectivity, excitatory/inhibitory neurotransmitter balance, or other factors). A recent study noted a modest correlation between increased MTL volume and summed durations of all seizures during ECT course; however, it is difficult to dissociate the number of treatments in each patient’s treatment course from this summed metric [42]. Regardless of the clinical utility of predictive models for seizure length, future studies seeking to further explain variance in seizure duration could still yield insight into the neurobiological nature of therapeutic seizure in ECT.

## Conclusions

This study identified instances where brain morphology influenced ECT-induced seizure. Seizures were assessed indirectly using the amount of electrical stimulation given (charge), the amount of stimulation reaching different brain areas (|E|), and seizure duration. Though we used these data to infer how seizure activity progresses through the brain during ECT, we have not measured this directly, an important limitation to consider. However, ictal neuroimaging also has limitations: molecular imaging lacks temporal resolution, scalp EEG lacks spatial resolution (particularly in deep MTL structures), and intracranial EEG lacks whole-brain coverage. As such, there is immense value in retrospective analysis of existing ECT-MRI datasets [99], and converging evidence across approaches will ultimately propel the field forward. Complementary efforts to measure seizure expression beyond duration will also be vital [20, 46, 100]. Additional limitations of the current study include cross-site differences in clinical procedures, limitations in available data due to the retrospective nature of the study, and the reliability [101] and/or fidelity of morphometric atlases used. Indeed, important structures are omitted by the current approach, including cerebellum, piriform, brainstem nuclei and smaller cortical subregions. Nevertheless, we expect these findings will inform future efforts to test (or refute) these ideas (e.g., whether site(s) of seizure initiation differ during seizure titration vs. supra-threshold stimulation, whether brain morphology can be used in models of personalized dosing). Future studies examining the combined contributions of brain morphometry, brain function, clinical and demographic information, and other factors may be best suited to develop personalized predictive models of ECT dose.

## Supplementary information

Supplemental Material

## Data Availability

Selected data for cohorts UMN2 and UCLA2 are available in NIMH Data Archive projects #2543 and #2844, respectively.
