# Supplementary material for: Brain morphometry, stimulation charge, and seizure duration in electroconvulsive therapy
Source: Mol Psychiatry. 2025 Nov 21;31(4):2094–105. doi: 10.1038/s41380-025-03360-y (PMC12999522; doi:10.1038/s41380-025-03360-y)
Supplement: Supplementary file 1 — Supplemental Material [file 41380_2025_3360_MOESM1_ESM.pdf]

Supplement: Brain morphometry, seizure duration, and stimulation charge in electroconvulsive therapy

Amber M. Leaver,* Chris Abbott, Randall Espinoza, Katherine L. Narr

Supplemental Methods

Data Selection. Once combined, the original source datasets included 199 total participants with MRI, demographic, and clinical data. Of these, 4 did not receive right unilateral ECT, an additional 26 had missing or incomplete ECT treatment data, and a further 3 had stimulation charge values that were 3 standard deviations above or below the mean for each of the first three ECT sessions; data from these participants were omitted from further analysis. Data from the remaining n=166 were included in analyses as reflected in **Table 1**. Additional exclusions were made for single statistical tests based on MRI QC procedures per metric, or because ECT treatment data were missing from ECT1 or ECT2&3 (not both). For UCLA1 cohort, stimulus charge and seizure duration were available only for the first three ECT sessions and the final ECT session, which in part informed our decision to study ECT1 and ECT2&3. Degrees of freedom are reported for each statistical analysis and reflect sample size (and regressors) used for each reported result.

ECT Parameters Note. ECT stimulation was comprised of pairs of electrical pulses, delivered at a given amplitude and frequency and repeated to form a “train” of pulses. If stimulation charge or one of its components was not recorded for a given ECT session, the following formula was used to calculate the missing value:

$$\text{Charge} = \text{amplitude} \times \text{pulse width} \times \text{frequency} \times \text{duration} \times 2$$

In this formula, the duration is the train duration, i.e., the total stimulation time. Impedance recordings were not available for ECT sessions in these datasets.

MRI Parcellated Whole-Brain Analysis. As noted in the main manuscript, all MRI metrics (gray matter volume, white matter volume, cortical thickness, cortical area, cortical curvature, and |E|) were averaged within parcels using standard Freesurfer atlases. This approach offers improved signal-to-noise over voxel- or vertex-wise analyses, though it may be less sensitive to focal effects.

Statistics, Significance & Effect Size. In main statistical analyses, metrics reflecting macro-anatomical structures (**Table S4**) were reported at a statistical criterion of uncorrected $p < 0.05$, and statistics for all other metrics were reported with correction for multiple comparisons using the false discovery rate (FDR) $q < 0.05$ (Benjamini-Hochberg method), applied separately for each model and metric type. For main analyses of |E|, we report results meeting both FDR $q < 0.05$ for $|E|_{verum}$ and uncorrected $p < 0.05$ for $|E|_{standard}$ (i.e., a conjunction of these two criteria), because stimulation amplitude contributes both to $|E|_{verum}$ and modestly to stimulation charge. Effect size is reported as partial r^2 (partial_r2 function, (26)), an estimate of the unique variance explained by each model term.

Statistics, Follow-up Analyses. Follow-up analyses were performed to aid in interpretation of the main analyses of stimulation charge, and to address potential cross-site differences in ECT administration (e.g., experimental manipulation of amplitude in the UNM2 cohort). Follow-up analyses were performed for each main analysis result for stimulation charge meeting statistical criteria as described above (for main analyses). First, separate statistical models replaced explained variable charge with either number of pulses or amplitude. Predictor variable of interest was MRI metric or $|E|_{standard}$, and regressors of no interest were age, sex, cohort, and amplitude (when pulse number was the explained variable) or pulse number (when amplitude was the explained variable). Our hypothesis was that these models would be $p < 0.05$ for pulse number, but not amplitude. Datapoints with pulse width = 1.0 from UNM2 were excluded (n=12) (1). In a second set of follow-up analyses, statistical models were applied separately for UNM2 and UCLA cohorts. Models were constructed as in the main analysis, with charge as the dependent variable, MRI metric or |E| as the regressor of interest, and age and sex as regressors of no interest. Results for follow-up analyses are reported uncorrected $p < 0.05$.

Additional follow-up analyses determined whether regional metrics (morphometry or $|E|_{standard}$) identified in the main analyses differed between responders and nonresponders to RUL ECT. Here, response was defined as >50% improvement in Hamilton Depression Rating Scale (HDRS) score after RUL ECT, and nonresponse was defined as (1) < 50% improvement in HDRS score after RUL ECT, where only RUL treatments were given OR (2) transition to bitemporal (BT) ECT regardless of post-treatment HDRS score. Data for patients who did not complete ECT (Non-completers, NC) are displayed in figures, but were not included in these analyses. In these linear models, explained variable was MRI metric or $|E|_{standard}$, predictor variable of interest was response (categorical factor), and variables of no interest were age, sex, and cohort. Homoscedasticity of variance between groups was confirmed using Levene’s test (car library). Results are reported

uncorrected $p < 0.05$, and relationships amongst variables meeting this statistical criterion were compared pairwise using Pearson's correlation coefficient. In this analysis, a negative correlation between cortical surface area near the right temporal electrode and $|E|_{standard}$ in anterior medial temporal lobe structures was identified. A mediation analysis explored this result further as described in the next section.

Statistics, Mediation Analysis. A mediation analysis probed whether $|E|_{standard}$ in anterior medial temporal lobe (AMTL) regions mediated (explained) the relationship between cortical surface area and antidepressant response to RUL ECT (mediation package in R (27)). Linear models were used to remain consistent with other statistics reported in this manuscript. For the full model, dependent variable was response (binary, numeric), with response defined as >50% improvement in HDRS score and nonresponse <50% improvement in HDRS score or transition to BT ECT. Independent variables were surface area of the anterior insula, $|E|_{standard}$ averaged across all AMTL regions identified in the follow-up analysis of response, as well as age, sex, and cohort. For the mediation model, dependent variable was mean AMTL $|E|_{standard}$ (mediator), and independent variables were anterior insula surface area (treatment), age, sex, and cohort. The mediate function was used, with confidence intervals estimated with nonparametric bootstrapping using the percentile method (5000 simulations). Surface area and $|E|_{standard}$ were z-normalized prior to this analysis.

Supplemental Results

ECT Parameters. Because UNM2 cohort participants were randomly assigned to 600, 700, or 800mA stimulation amplitude, additional analyses assessed relationships between stimulation charge, amplitude, and other components of stimulation dose in each cohort separately. The rationale was to demonstrate that charge was not simply a recapitulation of amplitude. In these analyses, stimulation charge and amplitude were not strongly related in the UNM2 cohort ($r = -0.23$ in ECT1 and $r = -0.34$ in ECT2&3, $p > 0.05$ for both; **Figure S1**). Instead, stimulation charge was positively correlated with paired-pulse train duration ($r = 0.395$ for ECT1; $r = 0.928$ for 2&3) and pulse width ($r = 0.725$ for ECT1; $r = 0.628$ for ECT2&3) in the UNM2 cohort ($p < 0.05$ for all; **Figure S1)**. This suggests that the higher range of stimulation charge in the UNM2 cohort was not explained well by amplitude, but instead reflects the combination of multiple stimulus parameters, just as in other cohorts. Additional descriptive statistics for these ECT parameters can be found in **Table 1** and **Table S1**.

Though charge (and number of pulses) during early sessions was marginally higher in participants who did not respond to RUL ECT (including transition to BT ECT), these differences appeared to be driven by outliers and were not considered further in the current manuscript (**Table S3; Figure S2**). Seizure duration did not differ in people who did not respond to RUL ECT (**Table S3; Figure S2**).

Supplemental Discussion

Regarding Seizure Titration

During the first ECT session, each patient's seizure threshold is estimated, and electrical dose on subsequent sessions is increased in reference to this threshold estimate (e.g., 6x seizure threshold in RUL ECT). In the titration method, a low dose of stimulation is incrementally increased until a seizure of desired morphology occurs; however, seizure threshold can also be estimated, albeit crudely, using the patient's age, sex, and/or past history with ECT (28,29). In the present study, the range of stimulation charge was wider for UNM cohorts during the first session, reflecting the experimental dose titration procedure used in the UNM2 cohort (1). The UCLA cohorts had a narrower range of stimulation charge on first session, where seizure titration followed the recommended schedule (protocol) for the MECTA device (Spectrum Q 100 Joule Titration Table for Ultrabrief 0.3ms Pulsewidth, (30)). Here, 80% of UCLA patients had threshold at step #2 (i.e., 20Hz frequency, 0.3ms width, 2s duration; **Figure S1**).

There are advantages and disadvantages to these approaches. Though seizure titration may provide a more accurate, individualized estimate of seizure threshold, excess stimulation may be a concern, e.g., if multiple rounds are needed to elicit seizure. Beginning titration with a higher dose more likely to elicit a seizure minimizes this risk; however, this method may over-estimate dose required for subsequent sessions (31), potentially increasing risk for cognitive or other side effects. Nevertheless, in the present study, stimulation dose at first session was much less than the dose used in subsequent sessions, and therefore first-session dose is likely to be much nearer to a patient's seizure threshold, even if titration methods differed across sites.

Mid-Hippocampus and ECT2&3 Charge

We interpret correlation between mid-hippocampal volume and ECT2&3 charge as reflecting its role in seizure generalization, perhaps via connections with thalamus. However, other interpretations are possible. Plasticity in mid-hippocampus after ECT has been linked with antidepressant response (32,33), so it is possible that the relationship between the size of this region and stimulation charge also reflects the importance of this region to antidepressant response. However, pre-treatment mid-hippocampus volume did not differ in nonresponders to RUL ECT in the current study (though when nonresponders did not include people who transitioned to BT ECT, mid-hippocampal volume was modestly higher in people who completed but did not respond to RUL ECT, similar to (4)). Though not measured in the current study, post-ECT plasticity may be more relevant; Deng, Abbott, and colleagues reported that increased total hippocampal volume after ECT mediated the relationship between total hippocampal |E| and antidepressant response (7). However, it is also possible that the size/length of mid-hippocampus affects the position of anterior hippocampus and amygdala within the E-field distribution for RUL ECT, thus influencing susceptibility to therapeutic seizure and antidepressant response. Here, studies precisely measuring electrode position (or stimulation area) and resulting E field in relation to the position of AMTL structures in each patient could be informative.

|E| in Cortical White Matter

The primary effects of electrical stimulation in ECT and other brain stimulation technologies are generally assumed to occur in neuronal cell bodies and gray matter. However, other cells, including oligodendrocytes comprising white matter, astrocytes, interneurons, and even parts of the neurovascular unit (e.g., vascular smooth muscle) can all be influenced by electrical current (34–37). Indeed, this was the rationale for including both white and gray matter parcellations in our study. In addition, previous studies have also noted increased white matter integrity after ECT (38,39). Stimulation charge at both ECT1 and ECT2&3 correlated with |E| in white matter in right superior temporal cortex, as well as dorsal anterior cingulate and adjacent corpus callosum. Given that white matter is more conductive than gray matter, one possible interpretation of this finding is that electrical current (and thus seizure activity) may travel further from stimulating electrodes in patients with greater white matter |E|. For example, perhaps patients with more |E| reaching corpus callosum require less stimulation charge to elicit bilateral seizure activity at ECT1 (because the electrical stimulus is more likely to travel to the left hemisphere). Future studies incorporating EEG to measure seizure expression in right and left hemisphere in these patients could address this idea more directly.

ECT2&3 Seizure Duration and Subgenual Anterior and Posterior Cingulate Cortex (sgACC, PCC) Surface Area

Both the sgACC and PCC are implicated in self-referential processing and depressive neurobiology (40,41); however, it is unclear why surface area in these regions statistically predict seizure duration at supra-threshold stimulation in the current study. Though these metrics were not related to antidepressant response using our statistical criteria, pre-treatment surface area in sgACC was marginally smaller in responders to RUL ECT (t_{117}=1.93, p=0.06, partial r^2=0.03). In pairwise comparisons, sgACC surface area did not differ strongly between responders and nonresponders who continued RUL ECT (t_{150}=1.32, p=0.19, partial r^2=0.01) or transitioned to BT ECT (t_{150}=1.70, p=0.09, partial r^2=0.02). But, surface area for patients who did not complete ECT did appear to be larger compared with responders, though effect size was small (t_{150}=2.10, p=0.04, partial r^2=0.03). Given that patients with larger left sgACC tended to have longer seizures in this study, we speculate that these effects could be related to the size of nearby piriform cortex, which extends posteriorly from sgACC to entorhinal cortex and was not studied in this paper. Piriform cortex is a highly seizure-genic and understudied structure (42–44), and future studies directly measuring its contribution to ECT could be informative

Regarding the Selection of Stimulation Charge in Main Analyses

One of the goals of this study was to measure relationships between pre-treatment morphology and the amount of stimulation needed (or given) to elicit seizures during ECT. The main way we chose to measure the amount of stimulation given during treatment is stimulation charge (i.e., Total Charge in mC). Though stimulation charge is a summary measure combining multiple stimulation parameters, it has advantages. First, there is a history of using charge in previous ECT research, though its utility may be debated. Stimulation Charge is also a known/measured quantity (i.e., compared with |E|, which is estimated and not directly measured). Perhaps most importantly, using stimulation charge in this retrospective study may mitigate differences in stimulation parameters used across cohorts (e.g., experimental manipulation of amplitude in UNM2) and across patients and sessions (e.g., differences in pulse width in UNM2, or in other parameters for all patients). Systematic and independent experimental manipulation of each of these parameters would be the ideal way to assess their individual impacts; however, this is best suited to prospective trials, animal research, or even larger multi-cohort studies than the current study.

To complement our main analysis approach using stimulation charge, we also used follow-up analyses to address differences in amplitude across sites. These analyses confirmed that effects of charge were mostly consistent across sites, and were not driven by amplitude. Instead, these effects appeared to be better explained by pulse number (though

stimulation charge was typically still superior to pulse number). Though it was possible to calculate stimulation time instead of pulse number, the great majority of patients received ultra brief pulse stimulation (0.3s; n=154), and so these follow-up analyses omitted the few UNM2 patients who received longer pulse width stimulation as part of their clinical trial protocol (1.0s; n=12) and pulse number was analyzed.

Estimated magnitude of electrical current, or |E|, was another way to study the amount of stimulation given during treatment. Though a potentially powerful tool, there are limitations to estimating E-fields. As mentioned above, |E| is an estimate, rather than a direct measure of the electrical current reaching different head and brain tissues. The accuracy of this estimate depends on the fidelity of tissue segmentation, how well the resistance of each patient's head tissues matches values assumed by the software, quality of the input MRIs, and other factors. In addition, both $|E|_{verum}$ and stimulation charge are calculated using stimulation amplitude; requiring a conjunction of $|E|_{verum}$ pFDR < 0.05 and $|E|_{standard}$ $p < 0.05$ for statistical significance attempted to mitigate this issue. Despite these limitations, |E| provides unique information about how much electric current reaches different parts of the brain across different patients and continues to be a useful tool in brain stimulation research.

Supplemental References

1. Abbott CC, Quinn D, Miller J, Ye E, Iqbal S, Lloyd M, *et al.* (2021): Electroconvulsive Therapy Pulse Amplitude and Clinical Outcomes. *Am J Geriatr Psychiatry* 29: 166–178.
2. Peterchev AV, Rosa MA, Deng Z-D, Prudic J, Lisanby SH (2010): Electroconvulsive therapy stimulus parameters: rethinking dosage. *J ECT* 26: 159–174.
3. Peterchev AV, Deng Z-D, Sikes-Keilp C, Feuer EC, Rosa MA, Lisanby SH (2024, September 30): Optimal Frequency for Seizure Induction with Electroconvulsive Therapy and Magnetic Seizure Therapy. bioRxiv, p 2024.09.28.615333.
4. Joshi SH, Espinoza RT, Pirnia T, Shi J, Wang Y, Ayers B, *et al.* (2015): Structural plasticity of the hippocampus and amygdala induced by electroconvulsive therapy in major depression. *Biol Psychiatry* 79: 282–92.
5. Tozzi L, Anene ET, Gotlib IH, Wintermark M, Kerr AB, Wu H, *et al.* (2021): Convergence, preliminary findings and future directions across the four human connectome projects investigating mood and anxiety disorders. *NeuroImage* 245: 118694.
6. Abbott CC, Jones T, Lemke NT, Gallegos P, McClintock SM, Mayer AR, *et al.* (2014): Hippocampal structural and functional changes associated with electroconvulsive therapy response. *Transl Psychiatry* 4: e483.
7. Deng Z-D, Argyelan M, Miller J, Quinn DK, Lloyd M, Jones TR, *et al.* (2022): Electroconvulsive therapy, electric field, neuroplasticity, and clinical outcomes. *Mol Psychiatry* 27: 1676–1682.
8. Destrieux C, Fischl B, Dale A, Halgren E (2010): Automatic parcellation of human cortical gyri and sulci using standard anatomical nomenclature. *NeuroImage* 53: 1–15.
9. Fischl B, Salat DH, Busa E, Albert M, Dieterich M, Haselgrove C, *et al.* (2002): Whole brain segmentation: automated labeling of neuroanatomical structures in the human brain. *Neuron* 33: 341–355.
10. Fischl B, Dale AM (2000): Measuring the thickness of the human cerebral cortex from magnetic resonance images. *Proc Natl Acad Sci* 97: 11050–11055.
11. Dale AM, Fischl B, Sereno MI (1999): Cortical surface-based analysis. I. Segmentation and surface reconstruction. *NeuroImage* 9: 179–194.
12. Iglesias JE, Augustinack JC, Nguyen K, Player CM, Player A, Wright M, *et al.* (2015): A computational atlas of the hippocampal formation using ex vivo, ultra-high resolution MRI: Application to adaptive segmentation of in vivo MRI. *NeuroImage* 115: 117–137.
13. Saygin ZM, Kliemann D, Iglesias JE, van der Kouwe AJW, Boyd E, Reuter M, *et al.* (2017): High-resolution magnetic resonance imaging reveals nuclei of the human amygdala: manual segmentation to automatic atlas. *NeuroImage* 155: 370–382.
14. Thielscher A, Antunes A, Saturnino GB (2015): Field modeling for transcranial magnetic stimulation: A useful tool to understand the physiological effects of TMS? *2015 37th Annual International Conference of the IEEE Engineering in Medicine and Biology Society (EMBC)* 222–225.
15. Saturnino GB, Puonti O, Nielsen JD, Antonenko D, Madsen KH, Thielscher A (2019): SimNIBS 2.1: A Comprehensive Pipeline for Individualized Electric Field Modelling for Transcranial Brain Stimulation. In: Makarov S, Horner M, Noetscher G, editors. *Brain and Human Body Modeling: Computational Human Modeling at EMBC 2018*. Cham (CH): Springer. Retrieved January 22, 2021, from http://www.ncbi.nlm.nih.gov/books/NBK549569/
16. Kellner CH, Knapp R, Husain MM, Rasmussen K, Sampson S, Cullum M, *et al.* (2010): Bifrontal, bitemporal and right unilateral electrode placement in ECT: randomised trial. *Br J Psychiatry J Ment Sci* 196: 226–34.
17. Burger HC, Milaan JB van (1943): Measurements of the specific Resistance of the human Body to direct Current. *Acta Med Scand* 114: 584–607.

18. Lee WH, Lisanby SH, Laine AF, Peterchev AV (2017): Minimum Electric Field Exposure for Seizure Induction with Electroconvulsive Therapy and Magnetic Seizure Therapy. *Neuropsychopharmacol Off Publ Am Coll Neuropsychopharmacol* 42: 1192–1200.
19. Abbott CC, Miller J, Farrar D, Argyelan M, Lloyd M, Squillaci T, *et al.* (2024): Amplitude-determined seizure-threshold, electric field modeling, and electroconvulsive therapy antidepressant and cognitive outcomes. *Neuropsychopharmacology* 49: 640–648.
20. Fortin J-P, Parker D, Tunç B, Watanabe T, Elliott MA, Ruparel K, *et al.* (2017): Harmonization of multi-site diffusion tensor imaging data. *NeuroImage* 161: 149–170.
21. Johnson WE, Li C, Rabinovic A (2007): Adjusting batch effects in microarray expression data using empirical Bayes methods. *Biostat Oxf Engl* 8: 118–127.
22. Fortin J-P, Cullen N, Sheline YI, Taylor WD, Aselcioglu I, Cook PA, *et al.* (2018): Harmonization of cortical thickness measurements across scanners and sites. *NeuroImage* 167: 104–120.
23. Yu M, Linn KA, Cook PA, Phillips ML, McInnis M, Fava M, *et al.* (2018): Statistical harmonization corrects site effects in functional connectivity measurements from multi-site fMRI data. *Hum Brain Mapp* 39: 4213–4227.
24. Radua J, Vieta E, Shinohara R, Kochunov P, Quidé Y, Green MJ, *et al.* (2020): Increased power by harmonizing structural MRI site differences with the ComBat batch adjustment method in ENIGMA. *NeuroImage* 218: 116956.
25. Cetin Karayumak S, Bouix S, Ning L, James A, Crow T, Shenton M, *et al.* (2019): Retrospective harmonization of multi-site diffusion MRI data acquired with different acquisition parameters. *NeuroImage* 184: 180–200.
26. Cinelli C, Hazlett C (2020): Making Sense of Sensitivity: Extending Omitted Variable Bias. *J R Stat Soc Ser B Stat Methodol* 82: 39–67.
27. Tingley D, Yamamoto T, Hirose K, Keele L, Imai K (2014): mediation: R Package for Causal Mediation Analysis. *J Stat Softw* 59: 1–38.
28. Francis-Taylor R, Ophel G, Martin D, Loo C (2020): The ictal EEG in ECT: A systematic review of the relationships between ictal features, ECT technique, seizure threshold and outcomes. *Brain Stimulat* 13: 1644–1654.
29. Kellner CH, Pritchett JT, Beale MD, Coffey CE (1997): *Handbook of ECT*. Washington, D.C.: American Psychiatric Press.
30. MECTA LLC (1997): spECTrum: MECTA Instruction Manual: 100 Joules Domestic. MECTA Corporation.
31. Luccarelli J, McCoy TH, Seiner SJ, Henry ME (2021): Total charge required to induce a seizure in a retrospective cohort of patients undergoing dose titration of right unilateral ultrabrief pulse ECT. *J ECT* 37: 40–45.
32. Leaver AM, Vasavada M, Kubicki A, Wade B, Loureiro J, Hellemann G, *et al.* (2020): Hippocampal subregions and networks linked with antidepressant response to electroconvulsive therapy. *Mol Psychiatry* 1–12.
33. Leaver AM, Vasavada M, Joshi SH, Wade B, Woods RP, Espinoza R, Narr KL (2019): Mechanisms of Antidepressant Response to Electroconvulsive Therapy Studied With Perfusion Magnetic Resonance Imaging. *Biol Psychiatry* 85: 466–476.
34. Bahr-Hosseini M, Bikson M (2021): Neurovascular-modulation: A review of primary vascular responses to transcranial electrical stimulation as a mechanism of action. *Brain Stimulat* 14: 837–847.
35. Wischnewski M, Alekseichuk I, Opitz A (2023): Neurocognitive, physiological, and biophysical effects of transcranial alternating current stimulation. *Trends Cogn Sci* 27: 189–205.
36. Mohan UR, Watrous AJ, Miller JF, Lega BC, Sperling MR, Worrell GA, *et al.* (2020): The effects of direct brain stimulation in humans depend on frequency, amplitude, and white-matter proximity. *Brain Stimulat* 13: 1183–1195.
37. Liu A, Vöröslakos M, Kronberg G, Henin S, Krause MR, Huang Y, *et al.* (2018): Immediate neurophysiological effects of transcranial electrical stimulation [no. 1]. *Nat Commun* 9: 5092.
38. Lyden H, Espinoza RT, Pirnia T, Clark K, Joshi SH, Leaver AM, *et al.* (2014): Electroconvulsive therapy mediates neuroplasticity of white matter microstructure in major depression. *Transl Psychiatry* 4: e380.
39. Repple J, Meinert S, Bollettini I, Grotegerd D, Redlich R, Zaremba D, *et al.* (2020): Influence of electroconvulsive therapy on white matter structure in a diffusion tensor imaging study. *Psychol Med* 50: 849–856.
40. Mayberg HS, Liotti M, Brannan SK, McGinnis S, Mahurin RK, Jerabek PA, *et al.* (1999): Reciprocal Limbic-Cortical Function and Negative Mood: Converging PET Findings in Depression and Normal Sadness. *Am J Psychiatry* 156: 675–82.
41. Spreng RN, Grady CL (2010): Patterns of Brain Activity Supporting Autobiographical Memory, Prospection, and Theory of Mind, and Their Relationship to the Default Mode Network. *J Cogn Neurosci* 22: 1112–1123.
42. Borger V, Schneider M, Taube J, Potthoff A-L, Keil VC, Hamed M, *et al.* (2021): Resection of piriform cortex predicts seizure freedom in temporal lobe epilepsy. *Ann Clin Transl Neurol* 8: 177–189.
43. Piredda S, Gale K (1985): A crucial epileptogenic site in the deep prepiriform cortex. *Nature* 317: 623–625.
44. Steinbart D, Yaakub SN, Steinbrenner M, Guldin LS, Holtkamp M, Keller SS, *et al.* (2023): Automatic and manual segmentation of the piriform cortex: Method development and validation in patients with temporal lobe epilepsy and Alzheimer's disease. *Hum Brain Mapp* 44: 3196–3209.

Supplemental Tables

Table S1. ECT Stimulation Parameters, Descriptive Statistics

	ECT1	ECT2	ECT3	Average, All Treatments
Amplitude (600,700,800,900mA)	20,22,114,4	19,22,117,3	21,19,116,13	NR**
Pulse Width (0.3, 1.0ms)	147,12	146,14	145,12*	NR**
Frequency, Mean(SD) in Hz	20.2(4.5)	48.5(20.1)	50.2(22.2)	53.8(22.6)
Pulse Train Duration, Mean(SD) in s	2.7 (1.4)	6.4(1.5)	6.8(4.5)	6.7(2.2)
Charge, Mean(SD) in mC	26.5(15.0)	160.4(98.2)	168.8(104.0)	188.6(107.4)
Seizure Duration, Mean(SD) in s	73.9(33.5)	66.3(22.3)	56.6(21.4)	57.0(17.9)

**For ECT3, 16 participants received pulse width 0.25ms. **Not Reported, because these values were typically set prior to beginning treatment.*

Table S2, EEG Duration x Stimulation Charge controlling for age, sex, cohort

		t	df	p	β	βse	partial r^2
Seizure Duration, ECT1	charge	-1.60	152	0.1111	-0.29	0.18	0.017
	age	-2.00	152	0.0476	-0.40	0.20	0.026
	sex	-1.89	152	0.0601	-10.29	5.43	0.023
	cohortUCLA2	0.53	152	0.5991	3.44	6.54	0.002
	cohortUNM1	-1.27	152	0.2059	-17.85	14.05	0.011
	cohortUNM2	-0.63	152	0.5322	-4.78	7.63	0.003
Stimulation Charge, ECT1	EEGduration	-1.60	152	0.1111	-0.06	0.04	0.017
	age	1.39	152	0.1667	0.12	0.09	0.013
	sex	-2.96	152	0.0036	-6.92	2.34	0.054
	cohortUCLA2	0.61	152	0.5407	1.75	2.86	0.002
	cohortUNM1	-0.33	152	0.7449	-2.02	6.18	0.001
	cohortUNM2	3.02	152	0.0030	9.81	3.25	0.057
Seizure Duration, ECT2&3	charge	-1.82	153	0.0714	-0.04	0.02	0.021
	age	-2.57	153	0.0112	-0.30	0.12	0.041
	sex	-1.14	153	0.2551	-3.58	3.13	0.008
	cohortUCLA2	1.04	153	0.3009	4.00	3.86	0.007
	cohortUNM1	-1.92	153	0.0569	-15.82	8.25	0.023
	cohortUNM2	0.12	153	0.9011	0.57	4.54	0.000
Stimulation Charge, ECT2&3	EEGduration	-1.82	153	0.0714	-0.55	0.31	0.021
	age	1.54	153	0.1259	0.69	0.45	0.015
	sex	-1.13	153	0.2615	-13.48	11.96	0.008
	cohortUCLA2	1.79	153	0.0752	26.17	14.61	0.021
	cohortUNM1	-0.86	153	0.3891	-27.44	31.76	0.005
	cohortUNM2	3.37	153	0.0009	56.38	16.71	0.069
Seizure Duration, Sessions 2+	charge	-3.97	159	0.0001	-0.05	0.01	0.090
	age	-2.22	159	0.0279	-0.21	0.09	0.030
	sex	-1.97	159	0.0508	-4.93	2.50	0.024
	cohortUCLA2	-0.66	159	0.5086	-2.09	3.16	0.003
	cohortUNM1	-2.35	159	0.0200	-15.87	6.75	0.034
	cohortUNM2	-1.32	159	0.1903	-5.01	3.81	0.011
Stimulation Charge, Sessions 2+	EEGduration	-3.97	159	0.0001	-1.73	0.44	0.090
	age	0.99	159	0.3237	0.54	0.55	0.006
	sex	-3.01	159	0.0030	-42.76	14.18	0.054
	cohortUCLA2	1.62	159	0.1081	29.19	18.06	0.016
	cohortUNM1	-0.11	159	0.9130	-4.32	39.52	<0.001
	cohortUNM2	4.62	159	<0.0001	95.55	20.67	0.118

Table S3, Responder vs. Nonresponder differences controlling for age, sex, cohort

	t	df	p	β	βse	partial_r2
ECT1 Charge	1.86	116	0.065	0.006	0.003	0.029
ECT1 EEGduration	1.40	116	0.164	0.002	0.001	0.017
ECT1 # of Pulses	2.49	113	0.014	0.004	0.002	0.052
ECT2&3 Charge	1.75	115	0.083	0.001	0.001	0.026
ECT2&3 EEGduration	0.38	115	0.706	0.001	0.002	0.001
ECT2&3 # of Pulses	2.03	114	0.045	0.001	0.000	0.035

Table S4. Macro-morphometry

Metric, Session	Measure	t	df	p	β	βse	partial r^2
Seizure Duration, ECT1	Total Intracranial Volume	2.20	151	0.03	<0.0001	<0.0001	0.031
	L Cortical GMV	2.50	151	0.01	0.0004	0.0002	0.040
	R Cortical GMV	2.77	151	0.006	0.0004	0.0002	0.048
	Subcortical GMV	0.82	151	0.42	0.0005	0.0006	0.004
	L Cerebral WMV	0.32	151	0.75	<0.0001	0.0001	0.001
	R Cerebral WMV	0.53	151	0.60	0.0001	0.0001	0.002
	CSF	-1.02	151	0.31	-0.0105	0.0102	0.007
Stimulation Charge, ECT1	Total Intracranial Volume	2.38	151	0.02	<0.0001	<0.0001	0.036
	L Cortical GMV	2.62	151	0.01	0.0002	0.0001	0.044
	R Cortical GMV	2.77	151	0.006	0.0002	0.0001	0.048
	Subcortical GMV	1.64	151	0.10	0.0004	0.0003	0.017
	L Cerebral WMV	0.80	151	0.43	<0.0001	<0.0001	0.004
	R Cerebral WMV	0.81	151	0.42	<0.0001	<0.0001	0.004
	CSF	0.74	151	0.46	0.0033	0.0045	0.004
Seizure Duration, ECT2&3	Total Intracranial Volume	1.79	152	0.07	<0.0001	<0.0001	0.021
	L Cortical GMV	3.01	152	0.003	0.0003	0.0001	0.056
	R Cortical GMV	3.13	152	0.002	0.0003	0.0001	0.061
	Subcortical GMV	1.12	152	0.27	0.0004	0.0003	0.008
	L Cerebral WMV	0.21	152	0.83	<0.0001	0.0001	0.000
	R Cerebral WMV	0.45	152	0.66	<0.0001	0.0001	0.001
	CSF	-0.33	152	0.74	-0.0020	0.0060	0.001
Stimulation Charge, ECT2&3	Total Intracranial Volume	1.64	152	0.10	0.0001	<0.0001	0.017
	L Cortical GMV	2.37	152	0.02	0.0008	0.0003	0.035
	R Cortical GMV	2.42	152	0.02	0.0008	0.0003	0.037
	Subcortical GMV	2.48	152	0.01	0.0031	0.0013	0.039
	L Cerebral WMV	0.65	152	0.52	0.0001	0.0002	0.003
	R Cerebral WMV	0.60	152	0.55	0.0001	0.0002	0.002
	CSF	0.68	152	0.50	0.0156	0.0230	0.003

Abbreviations: gray matter volume, GMV; white matter volume, WMV; cerebrospinal fluid, CSF; left, L; right, R

Table S5. Regional morphometry and pulse number

		Number of Pulses						Amplitude					
Session	Region, Measure	t	df	p	β	βse	partial r^2	t	df	p	β	βse	partial r^2
ECT1	R Inferior Frontal Gyrus Opercular Area, Mean Curv	2.06	138	0.0411	507.2	246.0	0.030	-0.10	140	0.9198	-0.052	0.512	0.000
	R Inferior Temporal Sulcus, SA	3.57	138	0.0005	0.050	0.014	0.084	-1.55	140	0.1232	0.000	0.000	0.017
	L Paracentral Gyrus and Sulcus, SA	2.62	138	0.0098	0.051	0.020	0.047	0.61	140	0.5417	0.000	0.000	0.003
	R Circular Sulcus, Superior Insula, SA	1.07	138	0.2857	0.023	0.022	0.008	0.48	140	0.6326	0.000	0.000	0.002
	R Posterior Lateral Fissure, SA	0.78	138	0.4369	0.019	0.024	0.004	0.53	140	0.5971	0.000	0.000	0.002
	L Superior Frontal Gyrus, SA	1.95	138	0.0528	0.007	0.004	0.027	-1.02	140	0.3101	0.000	0.000	0.007
	R Inferior Frontal Gyrus Opercular Area, SA	1.40	138	0.1641	0.023	0.016	0.014	-0.42	140	0.6758	0.000	0.000	0.001
ECT2&3	R Short Insular Gyrus, Mean Curv	2.00	139	0.0469	1446.8	721.6	0.028	1.20	139	0.2338	0.344	0.288	0.010
	L Thalamus, LP nucleus, Vol	2.07	139	0.0406	1.106	0.535	0.030	-0.39	139	0.6937	0.000	0.000	0.001
	R Hippocampus Body, Dentate Gyrus et al., Vol	3.17	125	0.0019	2.186	0.690	0.074	0.08	125	0.9378	0.000	0.000	0.000
	R Thalamus, AV nucleus, Vol	1.16	139	0.2471	0.628	0.541	0.010	-0.64	139	0.5231	0.000	0.000	0.003
	R Hippocampus Body CA4 region, Vol	3.13	125	0.0022	2.513	0.804	0.073	0.41	125	0.6820	0.000	0.000	0.001
	R Thalamus, LP nucleus, Vol	1.82	139	0.0702	0.998	0.547	0.023	-0.38	139	0.7060	0.000	0.000	0.001
	L Thalamus, Inferior Pulvinar nucleus, Vol	1.70	139	0.0922	0.616	0.363	0.020	0.04	139	0.9687	0.000	0.000	0.000
	R Thalamus, VA nucleus, Vol	2.09	139	0.0380	0.475	0.227	0.031	0.46	139	0.6457	0.000	0.000	0.002
	R Thalamus, Total Vol	1.77	139	0.0794	0.027	0.015	0.022	-0.70	139	0.4842	0.000	0.000	0.004
	R Thalamus CeM nucleus, Vol	1.58	139	0.1157	1.631	1.030	0.018	0.58	139	0.5630	0.000	0.000	0.002

Abbreviations: right, R; left, L; curvature, Curv; surface area, SA; volume, Vol; lateral posterior, LP; anteroventral, AV; ventral anterior, VA; centromedian, CeM.

Table S6. Regional morphometry and stimulation charge, separate cohorts

		UNM2 Only						UCLA1 & UCLA2					
Session	Region, Measure	t	df	p	β	βse	partial r^2	t	df	p	β	βse	partial r^2
1	R Inferior Frontal Gyrus (Operculum), Mean Curv	3.87	51	0.0003	927.34	239.5	0.227	1.77	92	0.0799	192.89	108.9	0.033
	R Inferior Temporal Sulcus, SA	2.62	51	0.0114	0.041	0.016	0.119	3.25	92	0.0016	0.020	0.006	0.103
	L Paracentral Gyrus and Sulcus, SA	3.25	51	0.0021	0.066	0.020	0.171	1.57	92	0.1189	0.015	0.009	0.026
	R Circular Sulcus, Superior Insula, SA	3.62	51	0.0007	0.088	0.024	0.205	1.55	92	0.1237	0.015	0.010	0.026
	R Posterior Lateral Fissure, SA	3.01	51	0.0041	0.078	0.026	0.151	1.50	92	0.1366	0.016	0.011	0.024
	L Superior Frontal Gyrus, SA	3.06	51	0.0035	0.012	0.004	0.155	1.66	92	0.0997	0.003	0.002	0.029
	R Inferior Frontal Gyrus Opercular Area, SA	3.33	51	0.0016	0.064	0.019	0.178	1.13	92	0.2617	0.008	0.007	0.014
2&3	R Short Insular Gyrus, Mean Curv	2.85	51	0.0062	2270.9	795.9	0.138	2.51	93	0.0138	706.2	281.4	0.063
	L Thalamus, LP nucleus, Vol	2.35	51	0.0225	1.367	0.581	0.098	2.24	93	0.0273	0.529	0.236	0.051
	R Hippocampus Body, Dentate Gyrus et al., Vol	2.53	40	0.0155	2.622	1.037	0.138	2.09	91	0.0391	0.594	0.284	0.046
	R Thalamus, AV nucleus, Vol	3.68	51	0.0006	2.161	0.588	0.210	1.18	93	0.2425	0.255	0.217	0.015
	R Hippocampus Body CA4 region, Vol	2.31	40	0.0260	2.702	1.168	0.118	2.09	91	0.0393	0.695	0.332	0.046
	R Thalamus, LP nucleus, Vol	2.56	51	0.0136	1.594	0.623	0.114	1.34	93	0.1848	0.310	0.232	0.019
	L Thalamus, Inferior Pulvinar nucleus, Vol	2.49	51	0.0161	1.074	0.432	0.108	2.77	93	0.0068	0.383	0.139	0.076
	R Thalamus, VA nucleus, Vol	2.48	51	0.0166	0.753	0.304	0.107	2.88	93	0.0049	0.250	0.087	0.082
	R Thalamus, Total Vol	2.97	51	0.0046	0.051	0.017	0.147	1.71	93	0.0914	0.011	0.006	0.030
	R Thalamus CeM nucleus, Vol	2.59	51	0.0124	3.136	1.209	0.117	1.81	93	0.0729	0.769	0.424	0.034

Abbreviations: right, R; left, L; curvature, Curv; surface area, SA; volume, Vol; lateral posterior, LP; anteroventral, AV; ventral anterior, VA; centromedian, CeM.

Table S7. Estimated $|E|_{standard}$ and Pulse Number

		Number of Pulses						Amplitude					
Session	Region, Metric	t	df	p	β	βse	partial r^2	t	df	p	β	βse	partial r^2
1	R Superior Temporal WM	-2.38	137	0.019	-0.16	0.07	0.040	-0.76	137	0.450	0.00	0.00	0.004
1	R Inferior Temporal WM	-2.29	137	0.024	-0.20	0.09	0.037	-0.28	137	0.781	0.00	0.00	0.001
1	R Caudal Anterior Cingulate WM	-2.87	137	0.005	-0.25	0.09	0.057	-0.57	137	0.568	0.00	0.00	0.002
1	R Transverse Temporal WM	-1.96	137	0.052	-0.18	0.09	0.027	-0.88	137	0.381	0.00	0.00	0.006
2&3	R Amygdala, Corticoamygdaloid Transition	-1.46	124	0.146	-0.31	0.21	0.017	-0.32	124	0.749	0.00	0.00	0.001
2&3	R Hippocampus Head, HATA	-1.78	124	0.077	-0.35	0.19	0.025	-0.40	124	0.687	0.00	0.00	0.001
2&3	R Amygdala, Total	-1.84	137	0.069	-0.31	0.17	0.024	0.01	137	0.993	0.00	0.00	0.000
2&3	R Superior Temporal WM	-2.38	137	0.019	-0.16	0.07	0.040	-0.76	137	0.450	0.00	0.00	0.004
2&3	R Amygdala, Accessory Basal Nucleus	-1.11	124	0.268	-0.24	0.21	0.010	-0.05	124	0.959	0.00	0.00	0.000
2&3	R Hippocampus, Parasubiculum	-1.67	124	0.097	-0.36	0.22	0.022	0.08	124	0.939	0.00	0.00	0.000
2&3	R Amygdala, Basal Nucleus	-1.35	124	0.178	-0.27	0.20	0.015	0.18	124	0.854	0.00	0.00	0.000
2&3	R Transverse Temporal WM	-1.96	137	0.052	-0.18	0.09	0.027	-0.88	137	0.381	0.00	0.00	0.006
2&3	R Putamen	-1.81	137	0.072	-0.27	0.15	0.023	0.20	137	0.845	0.00	0.00	0.000
2&3	R Hippocampus, Total	-1.74	137	0.085	-0.29	0.17	0.022	-0.02	137	0.985	0.00	0.00	0.000
2&3	R Lateral Orbitofrontal WM	-1.82	137	0.072	-0.18	0.10	0.023	0.32	137	0.753	0.00	0.00	0.001
2&3	R Hippocampus Head, Dentate et al.	-1.73	124	0.085	-0.33	0.19	0.024	0.16	124	0.873	0.00	0.00	0.000
2&3	R Inf Lateral Ventricle	-1.31	137	0.192	-0.16	0.12	0.012	-0.02	137	0.981	0.00	0.00	0.000
2&3	R Globus Pallidus	-1.78	137	0.077	-0.23	0.13	0.023	0.22	137	0.828	0.00	0.00	0.000
2&3	R Hippocampus Head, CA3	-1.68	124	0.095	-0.31	0.19	0.022	-0.09	124	0.930	0.00	0.00	0.000
2&3	R Inferior Temporal WM	-2.29	137	0.024	-0.20	0.09	0.037	-0.28	137	0.781	0.00	0.00	0.001
2&3	R Caudal Anterior Cingulate WM	-2.87	137	0.005	-0.25	0.09	0.057	-0.57	137	0.568	0.00	0.00	0.002
2&3	R Amygdala, Paralaminar Nucleus	-1.54	124	0.127	-0.30	0.19	0.019	0.03	124	0.977	0.00	0.00	0.000
2&3	R Insula, WM	-1.82	137	0.071	-0.19	0.10	0.024	0.27	137	0.790	0.00	0.00	0.001
2&3	R Inferior Frontal (Pars Triangularis) WM	-2.11	137	0.037	-0.17	0.08	0.031	-0.05	137	0.957	0.00	0.00	0.000
2&3	R Entorhinal WM	-1.77	137	0.078	-0.20	0.11	0.022	-0.15	137	0.878	0.00	0.00	0.000
2&3	R Hippocampus Head, CA4	-1.59	124	0.115	-0.29	0.18	0.020	0.45	124	0.650	0.00	0.00	0.002
2&3	R Parahippocampal WM	-1.65	137	0.100	-0.25	0.15	0.020	-0.18	137	0.854	0.00	0.00	0.000
2&3	R Inferior Frontal (Pars Orbitalis) WM	-2.08	137	0.039	-0.18	0.09	0.031	0.25	137	0.806	0.00	0.00	0.000

2&3	R Rostral Middle Frontal WM	-1.65	137	0.100	-0.17	0.10	0.020	0.40	137	0.687	0.00	0.00	0.001
2&3	R Middle Temporal WM	-2.11	137	0.037	-0.16	0.08	0.031	-0.25	137	0.801	0.00	0.00	0.000
2&3	R Hippocampus Head, CA1	-1.45	124	0.150	-0.27	0.18	0.017	0.01	124	0.995	0.00	0.00	0.000
2&3	R Fusiform WM	-2.25	137	0.026	-0.31	0.14	0.036	-0.72	137	0.473	0.00	0.00	0.004
2&3	R Hippocampus Head, Molecular Layer	-1.58	124	0.116	-0.28	0.17	0.020	0.27	124	0.791	0.00	0.00	0.001
2&3	R Thalamus, Lateral Geniculate Nucleus	-1.11	137	0.268	-0.15	0.13	0.009	0.31	137	0.757	0.00	0.00	0.001
2&3	Pons	-1.42	137	0.159	-0.36	0.25	0.014	-0.23	137	0.820	0.00	0.00	0.000
2&3	R Inferior Frontal (Pars Opercularis)	-1.36	137	0.175	-0.11	0.08	0.013	0.49	137	0.623	0.00	0.00	0.002

Abbreviations: right, R; left, L; white matter, WM; anterior cingulate cortex, ACC.

Table S8. Estimated $|E|_{standard}$ and charge, separate cohorts

		UNM2						UCLA					
Session	Region, Metric	t	df	p	β	βse	partial r^2	t	df	p	β	βse	partial r^2
1	R Superior Temporal WM	-1.02	50	0.311	-0.11	0.11	0.021	-3.05	90	0.003	-0.10	0.03	0.094
1	R Inferior Temporal WM	-0.69	50	0.492	-0.10	0.14	0.010	-2.87	90	0.005	-0.13	0.04	0.084
1	R Caudal Anterior Cingulate WM	0.18	50	0.860	0.02	0.13	0.001	-4.09	90	0.000	-0.16	0.04	0.157
1	R Transverse Temporal WM	-0.45	50	0.651	-0.07	0.15	0.004	-3.08	90	0.003	-0.14	0.04	0.095
2&3	R Amygdala, Corticoamygdaloid Transition	-1.71	39	0.095	-2.66	1.55	0.070	-2.27	93	0.025	-0.93	0.41	0.053
2&3	R Hippocampus Head, HATA	-1.38	39	0.175	-1.90	1.37	0.047	-2.75	93	0.007	-1.04	0.38	0.075
2&3	R Amygdala, Total	-1.92	50	0.061	-2.78	1.45	0.069	-2.08	94	0.040	-0.77	0.37	0.044
2&3	R Superior Temporal WM	-1.80	50	0.078	-1.05	0.58	0.061	-2.05	94	0.043	-0.29	0.14	0.043
2&3	R Amygdala, Accessory Basal Nucleus	-1.53	39	0.135	-2.65	1.74	0.056	-2.33	93	0.022	-0.99	0.42	0.055
2&3	R Hippocampus, Parasubiculum	-2.52	39	0.016	-4.89	1.94	0.140	-1.21	93	0.231	-0.54	0.45	0.015
2&3	R Amygdala, Basal Nucleus	-1.95	39	0.059	-3.77	1.93	0.089	-1.80	93	0.074	-0.72	0.40	0.034
2&3	R Transverse Temporal WM	-1.00	50	0.321	-0.80	0.80	0.020	-2.63	94	0.010	-0.53	0.20	0.069
2&3	R Putamen	-1.08	50	0.285	-1.45	1.34	0.023	-2.68	94	0.009	-0.84	0.31	0.071
2&3	R Hippocampus, Total	-1.42	50	0.163	-2.15	1.52	0.039	-2.01	94	0.048	-0.74	0.37	0.041
2&3	R Lateral Orbitofrontal WM	-1.15	50	0.255	-0.91	0.79	0.026	-2.29	94	0.025	-0.51	0.23	0.053
2&3	R Hippocampus Head, Dentate et al.	-1.94	39	0.060	-3.61	1.86	0.088	-1.77	93	0.080	-0.68	0.38	0.033
2&3	R Inf Lateral Ventricle	-1.17	50	0.249	-1.17	1.00	0.027	-2.03	94	0.046	-0.56	0.27	0.042
2&3	R Globus Pallidus	-0.68	50	0.502	-0.77	1.14	0.009	-3.01	94	0.003	-0.81	0.27	0.088
2&3	R Hippocampus Head, CA3	-1.72	39	0.093	-2.97	1.72	0.071	-1.92	93	0.058	-0.74	0.39	0.038
2&3	R Inferior Temporal WM	-1.37	50	0.178	-1.06	0.77	0.036	-2.24	94	0.027	-0.43	0.19	0.051
2&3	R Caudal ACC WM	-0.62	50	0.537	-0.43	0.69	0.008	-3.85	94	0.000	-0.71	0.18	0.136
2&3	R Amygdala, Paralaminar Nucleus	-1.89	39	0.067	-3.41	1.81	0.084	-1.60	93	0.113	-0.63	0.40	0.027
2&3	R Insula, WM	-0.74	50	0.463	-0.69	0.94	0.011	-2.52	94	0.013	-0.56	0.22	0.063
2&3	R Inferior Frontal (Pars Triangularis) WM	-0.75	50	0.455	-0.53	0.70	0.011	-2.89	94	0.005	-0.52	0.18	0.082
2&3	R Entorhinal WM	-1.05	50	0.298	-0.98	0.94	0.022	-1.94	94	0.055	-0.47	0.24	0.039
2&3	R Hippocampus Head, CA4	-1.93	39	0.061	-3.50	1.82	0.087	-1.45	93	0.151	-0.54	0.38	0.022
2&3	R Parahippocampal WM	-0.84	50	0.403	-1.19	1.42	0.014	-2.54	94	0.013	-0.86	0.34	0.064

2&3	R Inferior Frontal (Pars Orbitalis) WM	-0.51	50	0.614	-0.36	0.71	0.005	-2.96	94	0.004	-0.56	0.19	0.085
2&3	R Rostral Middle Frontal WM	-0.36	50	0.722	-0.28	0.78	0.003	-2.93	94	0.004	-0.65	0.22	0.083
2&3	R Middle Temporal WM	-1.09	50	0.281	-0.74	0.68	0.023	-1.98	94	0.051	-0.33	0.17	0.040
2&3	R Hippocampus Head, CA1	-1.59	39	0.121	-3.07	1.93	0.061	-1.72	93	0.089	-0.63	0.36	0.031
2&3	R Fusiform WM	-0.52	50	0.605	-0.67	1.29	0.005	-2.35	94	0.021	-0.72	0.31	0.055
2&3	R Hippocampus Head, Molecular Layer	-1.58	39	0.121	-2.86	1.80	0.060	-1.58	93	0.117	-0.56	0.35	0.026
2&3	R Thalamus, Lateral Geniculate Nucleus	-0.32	50	0.748	-0.36	1.10	0.002	-2.60	94	0.011	-0.77	0.29	0.067
2&3	Pons	-0.70	50	0.490	-1.57	2.26	0.010	-2.32	94	0.023	-1.24	0.53	0.054
2&3	R Inferior Frontal (Pars Opercularis)	-0.52	50	0.603	-0.35	0.68	0.005	-2.04	94	0.045	-0.34	0.17	0.042

Abbreviations: right, R; left, L; white matter, WM; anterior cingulate cortex, ACC.

Table S9. Pairwise differences in responders vs. nonresponders to RUL ECT $p < 0.05$

		t	df	p	β	βse	partial r^2
RUL R vs. RUL NR	R Inferior Frontal G Opercular Area, SA	1.98	150	0.049	68.00	34.27	0.03
	R Circular S, Superior Insula, SA	1.50	150	0.136	38.346	25.600	0.015
	R Inferior Frontal G Opercular Area, Mean Curv	1.44	150	0.152	0.003	0.002	0.014
	R Amygdala, Paralaminar |E|	-2.34	136	0.020	-6.583	2.807	0.039
	R Hippocampus, Parasubiculum |E|	-2.08	136	0.039	-5.244	2.519	0.031
	R Hippocampus Head, Molecular Layer |E|	-1.98	136	0.049	-6.075	3.065	0.028
	R Amygdala, Basal Nucleus |E|	-2.04	136	0.044	-5.626	2.761	0.030
	R Hippocampus Head, CA1 |E|	-1.94	136	0.055	-5.713	2.947	0.027
	R Hippocampus Head, CA4 |E|	-1.56	136	0.122	-4.565	2.935	0.017
	R Hippocampus Head, Dentate et al. |E|	-1.54	136	0.126	-4.400	2.854	0.017
	R Amygdala, Accessory Basal Nucleus |E|	-1.69	136	0.093	-4.556	2.695	0.021
RUL R vs. xBT	R Inferior Frontal G Opercular Area, SA	2.12	150	0.035	61.03	28.75	0.03
	R Circular S, Superior Insula, SA	1.84	150	0.068	39.526	21.479	0.022
	R Inferior Frontal G Opercular Area, Mean Curv	2.83	150	0.005	0.005	0.002	0.051
	R Amygdala, Paralaminar |E|	-2.81	136	0.006	-6.904	2.456	0.055
	R Hippocampus, Parasubiculum |E|	-2.61	136	0.010	-5.758	2.203	0.048
	R Hippocampus Head, Molecular Layer |E|	-2.75	136	0.007	-7.364	2.681	0.053
	R Amygdala, Basal Nucleus |E|	-2.54	136	0.012	-6.129	2.415	0.045
	R Hippocampus Head, CA1 |E|	-2.63	136	0.009	-6.785	2.578	0.048
	R Hippocampus Head, CA4 |E|	-2.37	136	0.019	-6.090	2.567	0.040
	R Hippocampus Head, Dentate et al. |E|	-2.21	136	0.029	-5.514	2.497	0.035
	R Amygdala, Accessary Basal Nucleus |E|	-1.67	136	0.098	-3.930	2.357	0.020
RUL R vs. NC	R Inferior Frontal G Opercular Area, SA	2.17	150	0.031	69.23	31.84	0.03
	R Circular S, Superior Insula, SA	2.26	150	0.025	53.850	23.788	0.033
	R Inferior Frontal G Opercular Area, Mean Curv	2.38	150	0.018	0.005	0.002	0.037
	R Amygdala, Paralaminar |E|	-1.69	136	0.094	-4.563	2.704	0.021
	R Hippocampus, Parasubiculum |E|	-1.28	136	0.201	-3.116	2.426	0.012
	R Hippocampus Head, Molecular Layer |E|	-1.44	136	0.152	-4.252	2.952	0.015
	R Amygdala, Basal Nucleus |E|	-1.23	136	0.221	-3.269	2.659	0.011
	R Hippocampus Head, CA1 |E|	-1.23	136	0.222	-3.482	2.838	0.011
	R Hippocampus Head, CA4 |E|	-0.92	136	0.360	-2.597	2.826	0.006
	R Hippocampus Head, Dentate et al. |E|	-0.91	136	0.362	-2.512	2.749	0.006
	R Amygdala, Accessory Basal Nucleus |E|	-0.80	136	0.427	-2.069	2.595	0.005

Abbreviations: right, R; left, L; white matter, WM; surface area, SA; electric field magnitude, |E|; gyrus, G; sulcus, S; curvature, Curv; anterior cingulate cortex, ACC.

Figure S1

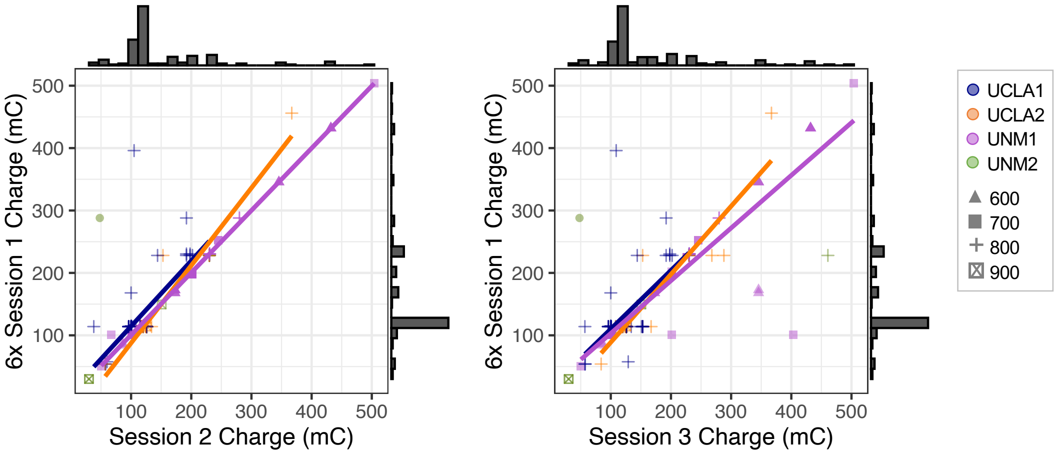

Figure S1. Relationships between six times (6x) ECT1 charge at seizure titration/threshold and charge during ECT2 (left) and ECT3 (right). These plots demonstrate changes in stimulation parameters across sessions for some patients. Color denotes cohort.

Figure S2

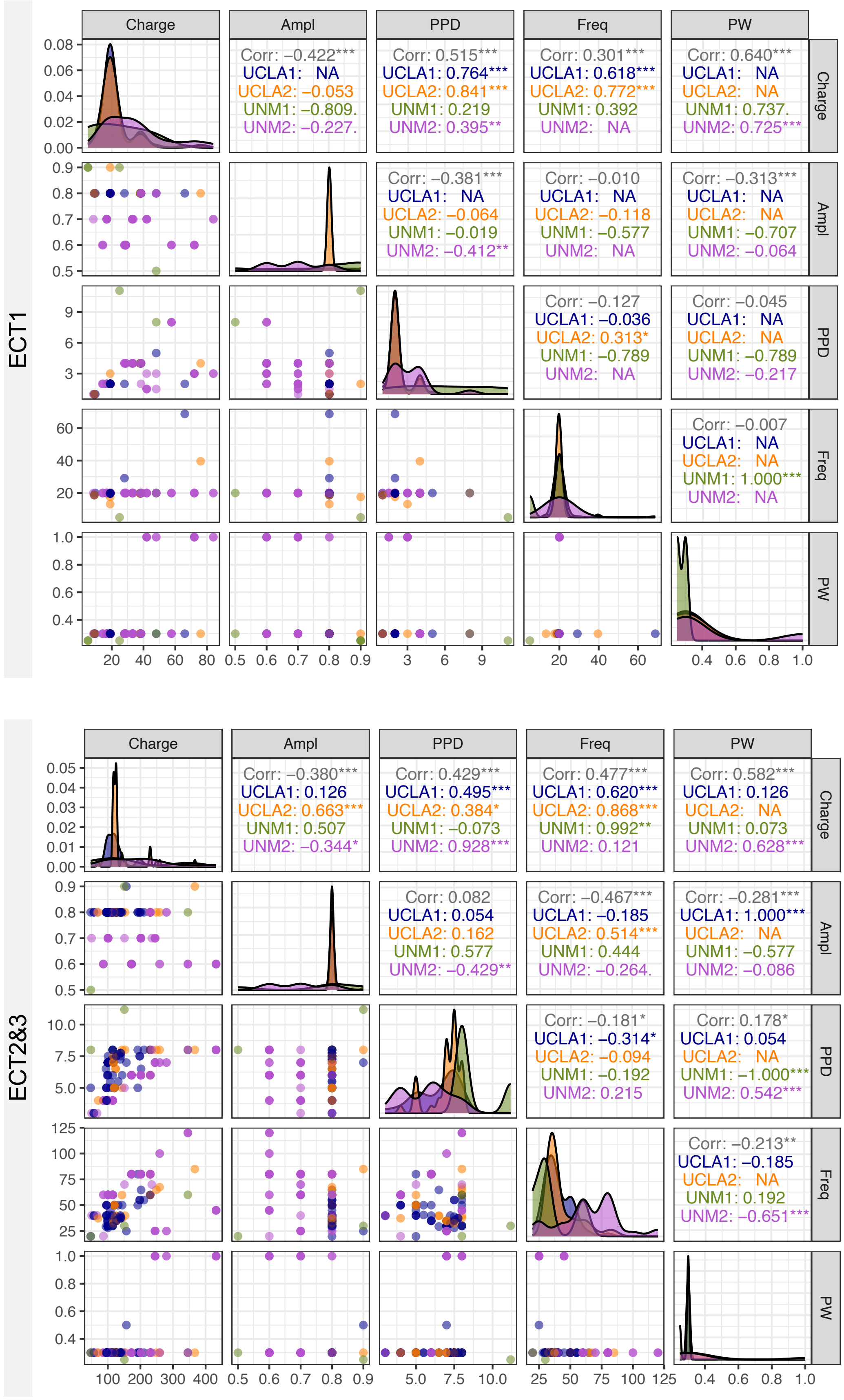

Figure S2. Relationships between each pair of ECT Parameters for ECT1 (top grid) and ECT2&3 (bottom grid) are are displayed. Lower left triangle of plots display scatter plots, upper right triangle display Pearson's r, and diagonal displays curved histograms for each metric. Color denotes cohort. ECT parameters include Charge (mC), Amplitude (Ampl, Amp), Pulse Train Duration (PPD, s), Frequency (Freq, Hz), and Pulse Width (PW, ms).

Figure S3

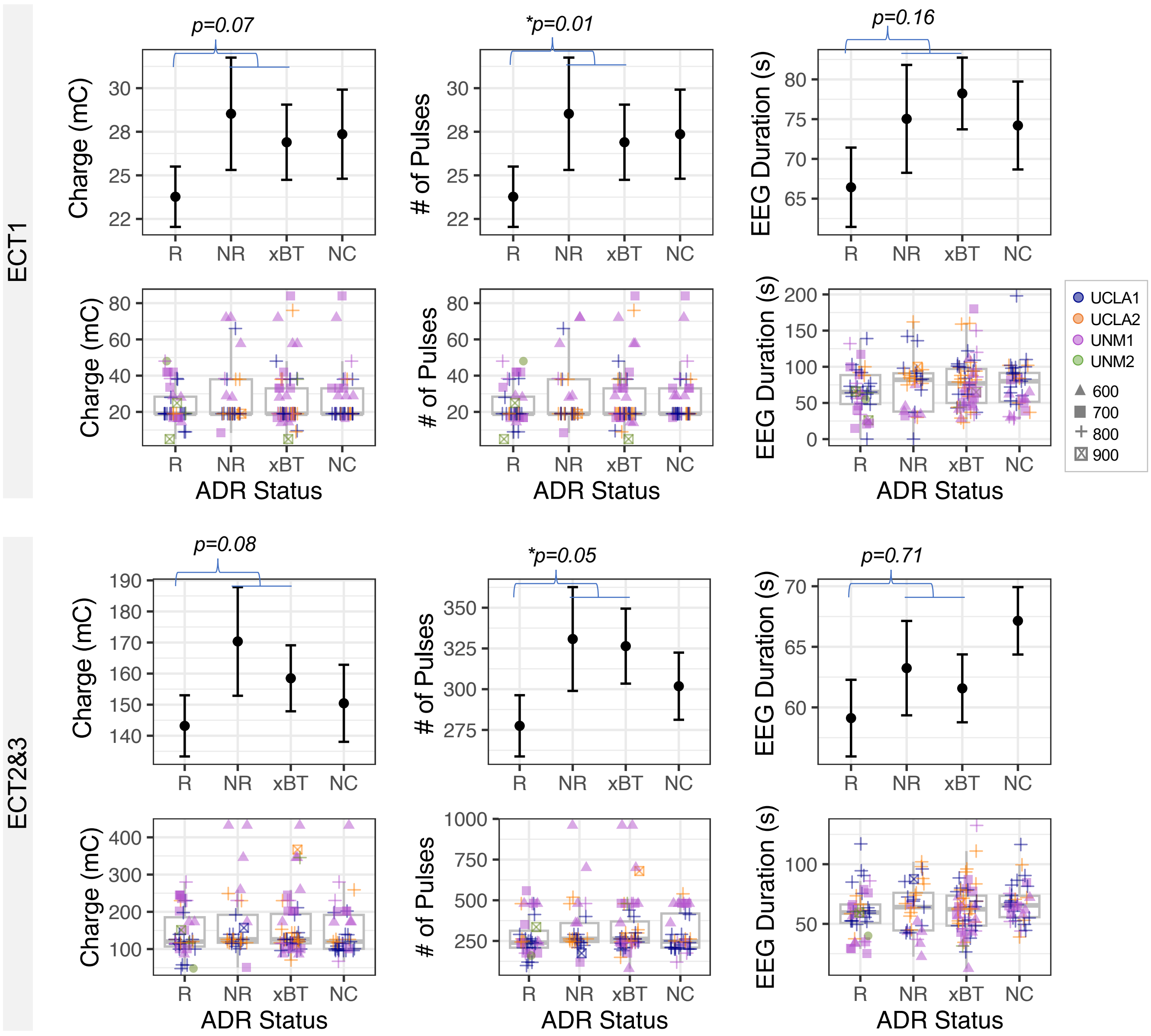

Figure S3. ECT Parameters for ECT1 (top 2 rows) and ECT2&3 (bottom 2 rows) are plotted against antidepressant response to RUL ECT. In first and third rows, mean and standard error are plotted for responders (R, >50% HDRS improvement), nonresponders (NR, <50% HDRS improvement), transition to bitemporal ECT (xBT), and non-completers (NC). In second and fourth rows, boxplots are displayed, along with datapoints for each participant. Color indicates cohort, and shape reflects amplitude.
